# Polymorphic factor H-binding activity of CspA protects Lyme borreliae from the host complement in feeding ticks to facilitate tick-to-host transmission

**DOI:** 10.1371/journal.ppat.1007106

**Published:** 2018-05-29

**Authors:** Thomas Hart, Ngoc Thien Thu Nguyen, Nancy A. Nowak, Fuming Zhang, Robert J. Linhardt, Maria Diuk-Wasser, Sanjay Ram, Peter Kraiczy, Yi-Pin Lin

**Affiliations:** 1 Department of Biological Science, State University of New York at Albany, Albany, New York, United States of America; 2 Division of Infectious Diseases, Wadsworth Center New York State Department of Health, Albany, New York, United States of America; 3 Institute of Medical Microbiology and Infection Control, University Hospital of Frankfurt, Frankfurt, Germany; 4 Division of Infectious Diseases and Immunology, University of Massachusetts Medical School, Worcester, Massachusetts, United States of America; 5 Department of Chemical and Biological Engineering, Rensselaer Polytechnic Institute, Troy, New York, United States of America; 6 Department of Chemistry and Chemical Biology, Rensselaer Polytechnic Institute, Troy, New York, United States of America; 7 Departments of Biology and Biomedical Engineering, Rensselaer Polytechnic Institute, Troy, New York, United States of America; 8 Department of Ecology, Evolution, and Environmental Biology, Columbia University, New York, New York, United States of America; 9 Department of Biomedical Science, State University of New York at Albany, Albany, New York, United States of America; University of Montana, UNITED STATES

## Abstract

*Borrelia burgdorferi* sensu lato (*Bbsl*), the causative agent of Lyme disease, establishes an initial infection in the host’s skin following a tick bite, and then disseminates to distant organs, leading to multisystem manifestations. Tick-to-vertebrate host transmission requires that *Bbsl* survives during blood feeding. Complement is an important innate host defense in blood and interstitial fluid. *Bbsl* produces a polymorphic surface protein, CspA, that binds to a complement regulator, Factor H (FH) to block complement activation *in vitro*. However, the role that CspA plays in the *Bbsl* enzootic cycle remains unclear. In this study, we demonstrated that different CspA variants promote spirochete binding to FH to inactivate complement and promote serum resistance in a host-specific manner. Utilizing a tick-to-mouse transmission model, we observed that a *cspA*-knockout *B*. *burgdorferi* is eliminated from nymphal ticks in the first 24 hours of feeding and is unable to be transmitted to naïve mice. Conversely, ectopically producing CspA derived from *B*. *burgdorferi* or *B*. *afzelii*, but not *B*. *garinii* in a *cspA*-knockout strain restored spirochete survival in fed nymphs and tick-to-mouse transmission. Furthermore, a CspA point mutant, CspA-L246D that was defective in FH-binding, failed to survive in fed nymphs and at the inoculation site or bloodstream in mice. We also allowed those spirochete-infected nymphs to feed on C3^-/-^ mice that lacked functional complement. The *cspA*-knockout *B*. *burgdorferi* or this mutant strain complemented with *cspA* variants or *cspA-L246D* was found at similar levels as wild type *B*. *burgdorferi* in the fed nymphs and mouse tissues. These novel findings suggest that the FH-binding activity of CspA protects spirochetes from complement-mediated killing in fed nymphal ticks, which ultimately allows *Bbsl* transmission to mammalian hosts.

## Author summary

Lyme disease, the most common vector-borne disease in the United States, is caused by the bacterium, *Borrelia burgdorferi*. This bacterium is transmitted to humans via the bite of a tick, and then spreads from the bite site to multiple tissues. Tick-to-human transmission of this bacterium requires bacterial survival in fed ticks because the blood and body fluid that ticks consume contain complement, an important mechanism that kills bacteria. To prevent host cell damage by this powerful mechanism, vertebrate animals produce factor H to inhibit complement. Lyme disease bacteria produce a surface protein CspA that binds to factor H to inhibit complement, but the role that CspA-mediated factor H-binding activity plays in tick-to-human transmission remains unexplained. To investigate this, we infected mice with Lyme disease strains that were identical except for the CspA variant or mutant with no factor H-binding ability they produced. We found that the factor H-binding activity of CspA appears to prevent the bacteria from being killed by complement in fed nymphal ticks. Such ability further facilitates bacterial transmission to mice. These results will promote the development of strategies against CspA to intervene in Lyme disease bacterial transmission from ticks to humans.

## Introduction

Lyme disease is caused by spirochetes of *Bbsl* and is transmitted to humans by the bites of infected *Ixodes* ticks. It is the most common vector-borne disease in North America and Europe [1, 2]. Upon blood feeding, spirochetes migrate from the ticks’ midguts to salivary glands, where they are transmitted to the host’s skin at the tick bite sites [2, 3]. In humans, Lyme borreliae initiate local skin infection often leading to erythema migrans, commonly known as a “bull’s-eye” rash [1, 2]. If left untreated, spirochetes are capable of entering the bloodstream and spreading to multiple tissues and organs, leading to arthritis, carditis, neuroborreliosis, and acrodermatitis chronica atrophicans [4]. The three main Lyme disease causing species, *B*. *afzelii*, *B*. *garinii*, and *B*. *burgdorferi* sensu stricto (hereafter *B*. *burgdorferi*), survive not only in humans, but also in other vertebrate animals [5]. These spirochete species tend to be associated with different vertebrate hosts: *B*. *afzelii* is typically isolated from small mammals, *B*. *garinii* from birds, and *B*. *burgdorferi* from both hosts [6, 7]. Specific spirochete-host associations are thought to be caused by the selective ability of these spirochetes to evade innate immune responses of different hosts. One such immune response, complement, is the first-line defense mechanism in humans and other vertebrates [7, 8].

The fluid-phase of complement is comprised of serum proteins, which are sequentially activated in response to invading pathogens [9, 10]. Complement can be initiated by three different pathways (the classical, alternative, and lectin pathways) which result in the formation of two distinct C3 convertases, C4b2a and C3bBb [11]. These C3 convertases recruit other complement components to generate C5 convertases, resulting in the release of pro-inflammatory peptides (C3a and C5a), the deposition of opsonins (C3b and iC3b) on microbial surfaces, and in the case of gram-negative organisms, lysis via insertion of the pore-forming membrane attack complex (C5b-9 also known as MAC) [11]. Humans and other vertebrates produce complement regulators to down-regulate the excessive complement activity to prevent host cell damage from non-specific attack by complement [9]. For example, factor H (FH) and factor H-like protein-1 (FHL-1, the spliced form of FH [12]) bind to C3b to promote its cleavage to iC3b, which is hemolytically inactive [9]. Interestingly, FH sequences from different vertebrate animals are diverse (e.g. 60 to 70% identity between mice and humans), suggesting a selective adaptation of FH to efficiently regulate the host-specific complement [13, 14].

Invading pathogens produce a variety of surface components such as capsules, lipopolysaccharides (e.g., O-antigens), and complement regulator-binding proteins to combat complement-mediated killing [15–18]. These complement regulator-binding proteins recruit these complement regulators to promote pathogen survival in the blood or serum (also known as serum resistance) [19, 20]. Like other pathogens, Lyme borreliae produce surface proteins that bind complement regulators to block the formation of lethal pores generated by the MAC. The resulting localization (and hence locally high concentration) of complement regulators on the spirochete surface permits survival of Lyme borreliae despite high concentrations of complement in the blood [21, 22]. *B*. *burgdorferi* produces five complement regulator acquiring surface proteins (CRASPs) including CspA (CRASP-1), CspZ (CRASP-2), ErpP (CRASP-3), ErpC (CRASP-4), and ErpA (CRASP-5) [23]. These CRASP proteins share activities in binding to FH (for all 5 CRASPs) and FHL-1 (for CspA and CspZ) [23] and degrading C3 or C5 convertases by binding to plasminogen (for all 5 CRASPs) [24, 25]. Additionally, CspA also inhibits complement by binding to C7 and C9 to block the formation of MAC [24, 25]. Unlike other CRASP genes, *cspA* is expressed when Lyme borreliae are in both fed and unfed nymphal ticks or at the inoculation site immediately after infection, suggesting that CspA may play a unique role in tick-to-mammal transmission [26, 27]. Ectopic production of CspA confers resistance to human serum in an *in vitro* gain-of-function study [24, 25], while deleting *cspA* from an infectious and serum-resistant *B*. *burgdorferi* strain makes this strain susceptible in human serum *in vitro* [28, 29]. These results indicate that CspA functions as a key factor to promote spirochete survival in serum.

CspA is highly conserved within *B*. *burgdorferi* (greater than 90% identity) but displays less than 50% sequence identity across different spirochete species [25]. Consistent with this variation, CspA variants from *B*. *burgdorferi*, *B*. *spielmannii*, or *B*. *afzelii* exhibit varying capacities to bind human FH and differ in their abilities to resist human serum [25, 30, 31]. These findings led us to hypothesize that the CspA-mediated FH-binding activity promotes spirochete serum survival in a vertebrate animal-specific manner. In addition, while the role that CspA plays *in vitro* has been extensively characterized, the function of this protein for Lyme borreliae in the enzootic cycle is still unclear. In this study, we elucidate the role of CspA-FH interactions in promoting complement evasion *in vitro* and tick-to-host transmission of Lyme borreliae.

## Results

### Polymorphic CspA variants have differential binding affinities to factor H from different vertebrate animals

The sequences of CspA variants among *B*. *burgdorferi* sensu lato are extremely polymorphic (S1 Fig) [25]. We thus sought to examine whether CspA polymorphisms account for variant-to-variant differences in their abilities to bind to FH from different vertebrate species. Thus, we tested the ability of recombinant CspA proteins derived from *cspA* sequences of *B*. *burgdorferi* strain B31 (CspA_B31_), *B*. *afzelii* strain PKo (CspA_PKo_), or *B*. *garinii* strain ZQ1 (CspA_ZQ1_) to bind to FH from different vertebrate animals. These animals include mouse (*Mus musculus*), *Coturnix* quail (rodent or avian model of Lyme disease [32, 33]), human (incidental host), and horse (dead-end host). We used ELISA and surface plasmon resonance (SPR) to evaluate the FH-binding affinity of CspA variants. A CspA mutant, CspA_B31_L246D, in which the leucine at position 246 was replaced by aspartic acid rendering it incapable of binding to human FH [34, 35], was included as negative control. As expected, while the irrelevant negative control protein *B*. *burgdorferi* DbpA did not bind to FH from these animals, both methods for assessing binding affinity indicated three distinct binding patterns of CspA variants in a host-specific manner (S2 and S3 Figs, and Table 1): (1) CspA_B31_ exhibited a versatile binding pattern to FH from all tested species (ELISA: K_D_ = 0.23–0.92 μM, SPR: K_D_ = 0.07–0.55 μM); (2) CspA_PKo_ possessed less flexible binding, with preference for human and mouse FH (ELISA: K_D_ = 0.16–0.76 μM, SPR: K_D_ = 0.11–0.46 μM), but not horse or quail FH; (3) CspA_ZQ1_ bound only to FH from quail (ELISA: K_D_ = 0.36 μM). As expected, the recombinant CspA_B31_L246D, which retained a secondary structure similar to wild type CspA_B31_ by far-UV CD analysis (S1 and S4 Figs and S1 Table), was unable to bind to FH from any of the tested species (S2 and S3 Figs and Table 1). These results indicate that CspA variants from different Lyme borreliae species bind variably to FH from different animals, and that leucine-246 of CspA_B31_ is essential for its ability to bind to FH from all tested animals.

**Table 1:** CspA variants differ in binding to factor H from different animals.

CspA variant	Factor H source	ELISA K_D_ (μM)^b^	-----Surface Plasmon Resonance----		
			K_D_ (μM)^a^	k_on_ (10^5^s^-1^M^-1^)	k_off_ (s^-1^)
***B*. *burgdorferi***					
**CspA**_**B31**_	**Human**	**0.41±0.09**	**0.20±0.07**	1.14±0.42	0.017±0.005
	**Mouse**	**0.23±0.08**	**0.07±0.01**	1.88±0.36	0.012±0.005
	**Horse**	**0.88±0.33**	**0.55±0.18**	0.30±0.01	0.014±0.003
	**Quail**	**0.92±0.28**	**n.d**.^a^	n.d.	n.d.
**CspA**_**B31**_**L246D**	**Human**	**n.s**.^b^	**n.b**.	n.b.	n.b.
	**Mouse**	**n.s**.	**n.b**.	n.b.	n.b.
	**Horse**	**n.b**.^c^	**n.b**.	n.b.	n.b.
	**Quail**	**n.b**.	**n.d**.	n.d.	n.d.
**DbpA**_**B31**_^d^	**Human**	**n.b**.	**n.d**.	n.d.	n.d.
	**Mouse**	**n.b**.	**n.d**.	n.d.	n.d.
	**Horse**	**n.b**.	**n.d**.	n.d.	n.d.
	**Quail**	**n.b**.	**n.d**.	n.d.	n.d.
***B*. *afzelii***					
**CspA**_**Pko**_	**Human**	**0.16±0.05**	**0.11±0.02**	1.52±0.62	0.014±0.004
	**Mouse**	**0.76±0.64**	**0.46±0.14**	1.15±0.31	0.048±0.011
	**Horse**	**n.b**.	**n.b**.	n.b.	n.b.
	**Quail**	**n.b**.	**n.d**.	n.d.	n.d.
***B*. *garinii***					
**CspA**_**ZQ1**_	**Human**	**n.b**.	**n.b**.	n.b.	n.b.
	**Mouse**	**n.b**.	**n.b**.	n.b.	n.b.
	**Horse**	**n.b**.	**n.b**.	n.b.	n.b.
	**Quail**	**0.36±0.13**	**n.d**.	n.d.	n.d.

All values represent the mean ± SEM of three experiments.

^a^Not determined.

^b^Not saturated

^c^No binding activity was detected.

^d^DbpA_B31_ was included as a negative control.

### Allelic variable CspA promotes spirochete binding to FH in a host-specific manner

**Fig 1:**
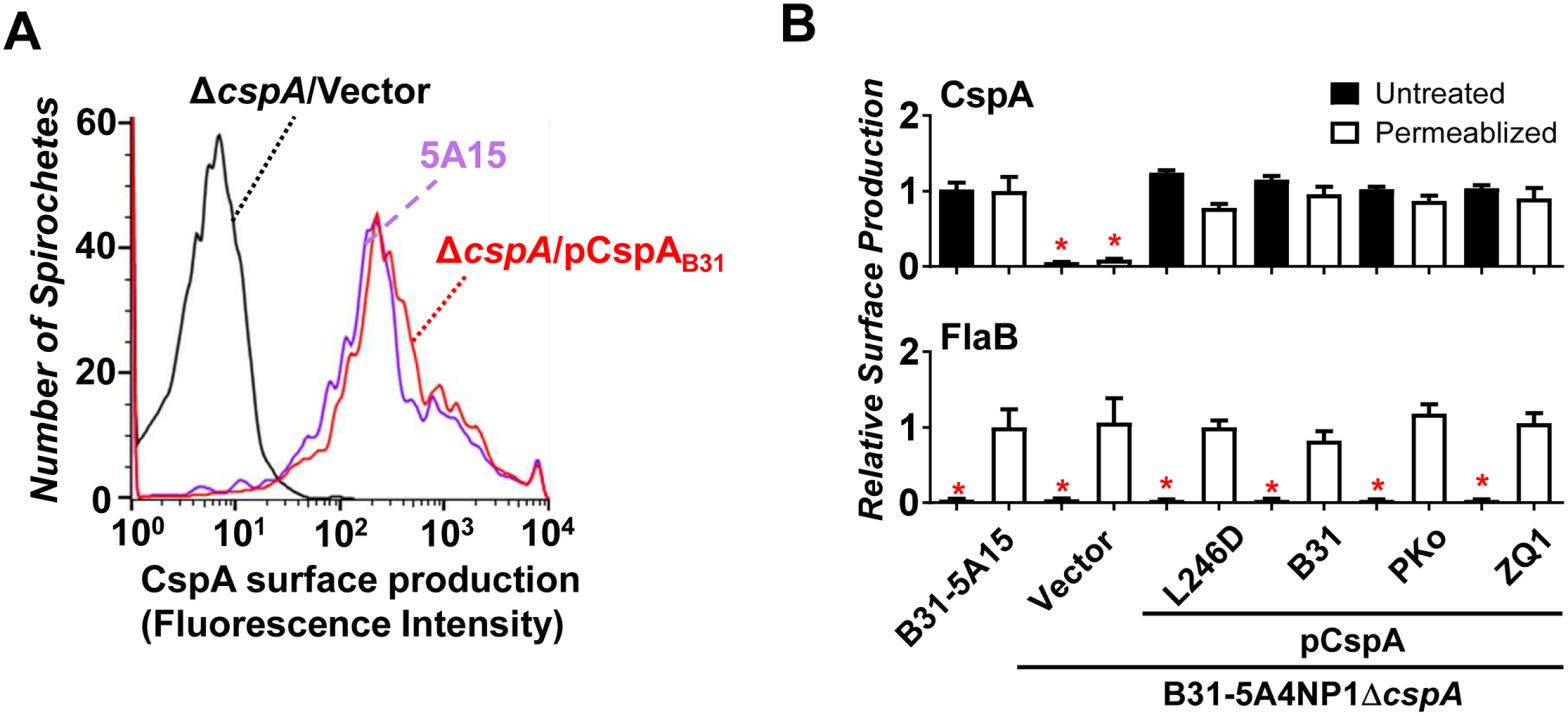
Localization of CspA on the surface of *B*. *burgdorferi*. Flow cytometry analysis of CspA localized on the surface of *B*. *burgdorferi* strains B31-5A15 (“B31-5A15”), B31-5A4NP1Δ*cspA* harboring the vector pBSV2G (“Δ*cspA*/Vector”), or this *cspA* mutant strain producing CspA_B31_ (“Δ*cspA*/pCspA_B31_”), CspA_PKo_ (“Δ*cspA*/pCspA_PKo_”), CspA_ZQ1_ (“Δ*cspA*/pCspA_ZQ1_”), or CspA_B31_L246D (“Δ*cspA*/pCspA_B31_L246D”). **(A)** Representative histograms of flow cytometry analysis showing the levels of CspA surface production to indicated *B*. *burgdorferi* strains. **(B)** The production of CspA and FlaB (negative control) on the surface of indicated *B*. *burgdorferi* strains was detected by flow cytometry (see Materials and methods). Values are shown relative to the production levels of CspA or FlaB on the surface of permeabilized *B*. *burgdorferi* strain B31-5A15. Each bar represents the mean of four independent determinations ± the standard deviation. (*): indicates that relative surface production of the indicated proteins was significantly lower (P < 0.05 by one-way ANOVA with post hoc Bonferroni correction) than that of CspA or FlaB by *B*. *burgdorferi* strain B31-5A15.

To examine whether the FH-binding characteristics of CspA proteins described above are maintained when these variants are produced on the surface of the spirochetes, we transformed shuttle plasmids encoding *cspA*_*B31*_, *cspA*_*PKo*_, *cspA*_*ZQ1*_, or *cspA*_*B31*_*L246D* under the control of the *cspA* promoter from *B*. *burgdorferi* strain B31 into *B*. *burgdorferi* strain B31-5A4NP1Δ*cspA* (5A4NP1Δ*cspA*), a *cspA* deficient strain previously generated from an infectious background *B*. *burgdorferi* strain B31-5A4NP1 (S2 Table) [28]. Because strain 5A4NP1Δ*cspA* was identified to have lost the plasmid lp21, *B*. *burgdorferi* strain B31-5A15 (B31-5A15), which has the same plasmid profile as strain 5A4NP1Δ*cspA* and is fully virulent, was used as a wild type (WT) positive control [28, 36]. We first used flow cytometry analysis to verify that the CspA variants or mutants produced in strain 5A4NP1Δ*cspA* are localized on the surface of spirochetes and at levels similar to the WT strain B31-5A15 (Fig 1).

**Fig 2:**
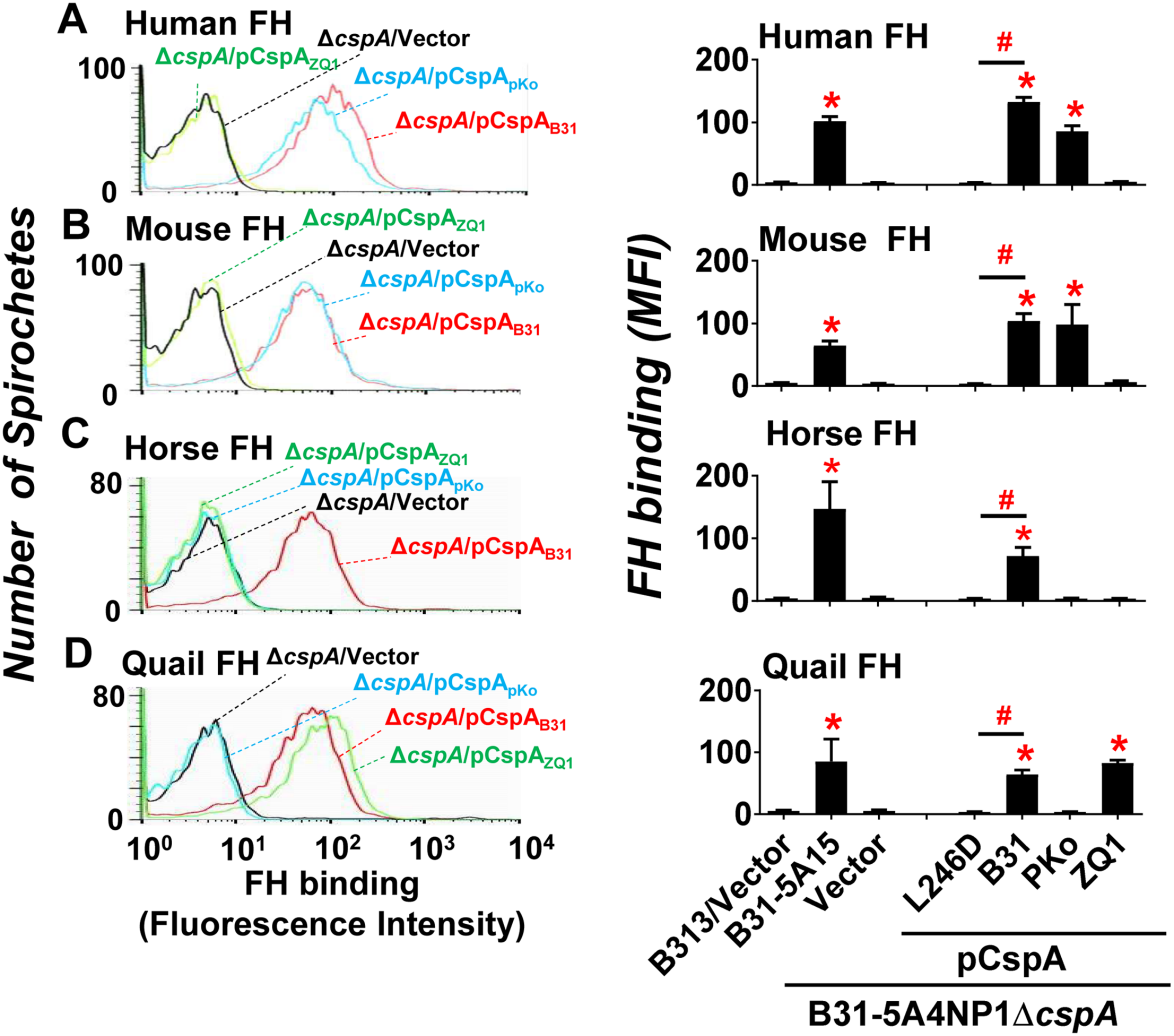
CspA variants differ in their ability in promoting spirochetes binding to FH from different vertebrate animals. *B*. *burgdorferi* strain B31-5A15 (“B31-5A15”), B31-5A4NP1Δ*cspA* harboring the vector pBSV2G (“Δ*cspA*/Vector”), or this *cspA* mutant strain producing CspA_B31_ (“Δ*cspA*/pCspA_B31_”), CspA_PKo_ (“Δ*cspA*/pCspA_PKo_”), CspA_ZQ1_ (“Δ*cspA*/pCspA_ZQ1_”), or CspA_B31_L246D (“Δ*cspA*/pCspA_B31_L246D”), or B313 carrying the vector pBSV2G (“B313/Vector”, negative control) was incubated with FH from human, mouse, horse, or quail. The bacteria were stained with a sheep anti-FH polyclonal IgG (for the spirochetes incubated with human, mouse, or horse FH) or a mouse anti-FH monoclonal antibody VIG8 (for the spirochetes incubated with quail FH) followed by an Alexa 647-conjugated donkey anti-sheep IgG or goat anti-mouse IgG prior to being applied to flow cytometry analysis. **(Left panel)** Representative histograms of flow cytometry analysis showing the levels of FH from **(A)** human, **(B)** mouse, **(C)** horse, or **(D)** quail binding to indicated *B*. *burgdorferi* strains. **(Right panel)** The levels of *B*. *burgdorferi* binding to FH from **(A)** human, **(B)** mouse, **(C)** horse, or **(D)** quail were measured by flow cytometry and presented as mean fluorescence index (MFI). Each bar represents the mean of three independent determinations ± SEM. Significant differences (P < 0.05 by one-way ANOVA with post hoc Bonferroni correction) in the levels of FH binding relative to the B313/Vector (“*”) or between two strains relative to each other (“#”).

We then incubated each of the CspA-producing 5A4NP1Δ*cspA*-derived strains, strain 5A4NP1Δ*cspA* harboring the vector alone (hereafter termed 5A4NP1Δ*cspA*-V), or the WT strain B31-5A15, with FH purified from human, mouse, horse, or quail. We then determined their FH-binding activity using flow cytometry. A high passage and non-infectious *B*. *burgdorferi* strain B313 (B313) harboring the shuttle vector alone was also included as a negative control as this strain does not encode *cspA* and does not bind human FH [37]. Consistent with previous findings [37], strain B313 (vector alone) did not bind human FH (Fig 2A right panel), or to FH from mouse, horse, and quail (Fig 2B to 2D right panel). The WT strain B31-5A15 bound FH from all these animals (Fig 2A to 2D right panel), in agreement with findings in previous studies [21, 28]. Strain 5A4NP1Δ*cspA*-V displayed undetectable levels of human FH-binding activity (Fig 2A), similar to previous observations [28]. Note that the infectious *B*. *burgdorferi* strain B31, the background strain of 5A4NP1Δ*cspA*-V, produces other human FH-binding proteins when cultivated *in vitro* [23, 26, 38]. However, these FH-binding proteins are either produced in extremely low levels [26] or display lower levels of FH-binding ability, compared to CspA [28, 39, 40]. These previous studies support our finding that nearly no human FH-binding activity was observed in this strain. Additionally, strain 5A4NP1Δ*cspA*-V did not show binding ability to mouse, horse, or quail FH (Fig 2B to 2D). These data indicate that CspA is essential for this infectious *B*. *burgdorferi* strain to bind to FH from all tested animals.

Further, expression of CspA variants in strain 5A4NP1Δ*cspA* restores the levels of binding to FH in a host-specific manner, reflecting the results obtained with the recombinant proteins (Fig 2): (1) The production of CspA_B31_ restored binding to FH from human, mouse, horse, and quail; (2) CspA_PKo_ increased binding to human and mouse FH but not to horse or quail FH; (3) CspA_ZQ1_ promoted binding only to quail FH but not to FH from other animals. Additionally, the *cspA*_*B31*_*L246D*-complemented strain showed no detectable binding to FH from all four animals tested (Fig 2A to 2D right panel). We also incubated the above-mentioned spirochete strains with C3-depleted human or mouse serum (C3-depleted horse or quail serum is not available) to verify the FH-binding activity in the context of serum components. C3-depleted serum was used because the strains that show reduced FH-binding would have greater levels of C3b deposited on their surfaces [28], which in turn would bind FH thereby confounding interpretation of results. We observed binding of human and mouse FH in C3 depleted sera as with purified FH (S5 Fig). These results indicate that the leucine-246 of this protein plays an essential role in facilitating spirochete binding to FH from these animals.

### Species to species variation of CspA-mediated FH binding contributes to different levels of complement deposition on the spirochete surface

**Fig 3:**
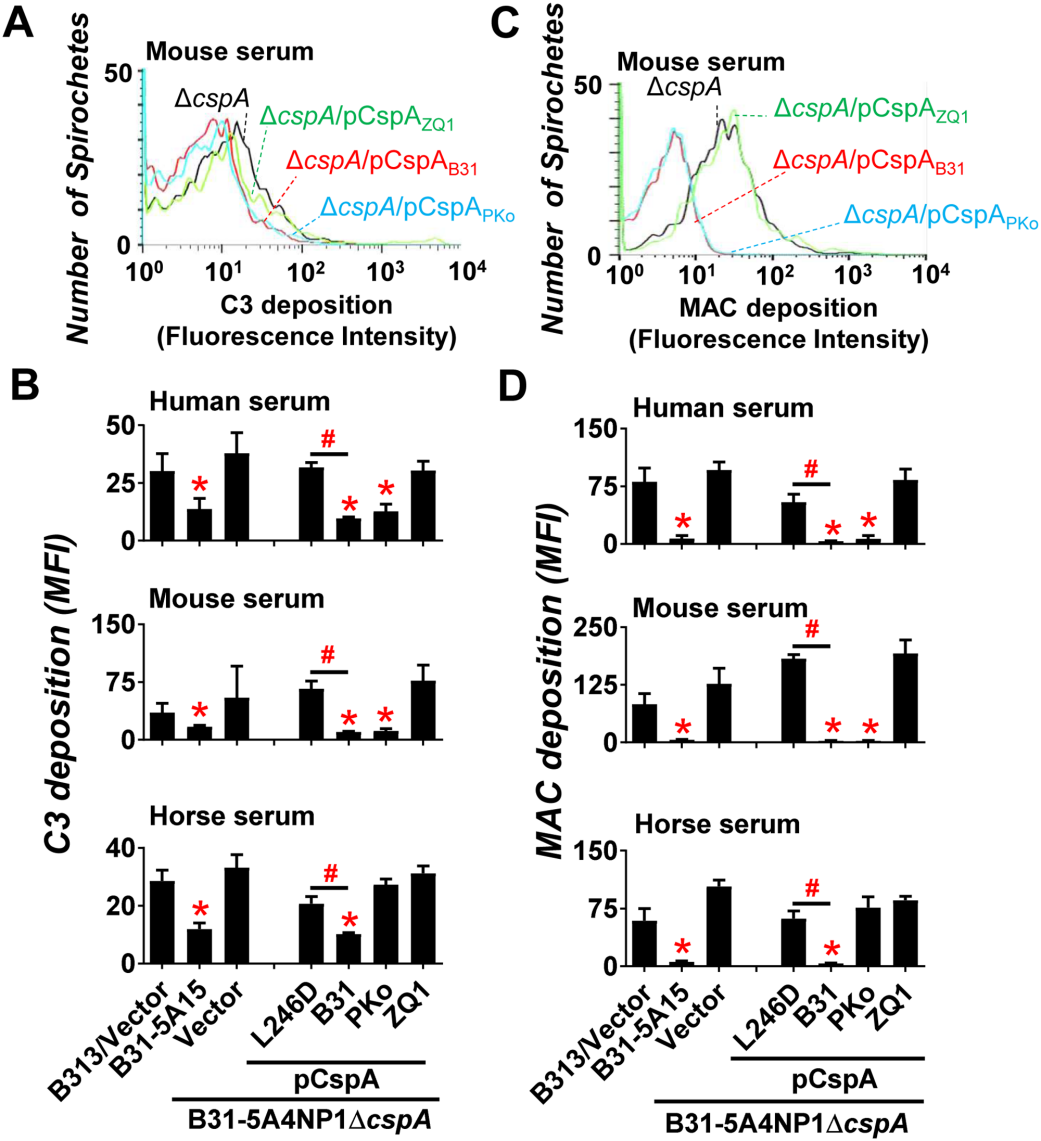
CspA variants differ in their ability to reduce the deposition of C3b or MAC from different vertebrate animals on the spirochete surface. *B*. *burgdorferi* strain B31-5A15 (“B31-5A15”), B31-5A4NP1Δ*cspA* harboring the vector pBSV2G (“Δ*cspA*/Vector”), or this *cspA* mutant strain producing CspA_B31_ (“Δ*cspA*/pCspA_B31_”), CspA_PKo_ (“Δ*cspA*/pCspA_PKo_”), CspA_ZQ1_ (“Δ*cspA*/pCspA_ZQ1_”), or CspA_B31_L246D (“Δ*cspA*/pCspA_B31_L246D”), or B313 carrying the vector pBSV2G (“B313/Vector”, negative control) was incubated with serum from human, mouse, or horse with a final concentration of 20%. The bacteria were stained with a guinea pig anti-C3 polyclonal IgG, a mouse anti-C5b-9 monoclonal antibody aE11 (for spirochetes incubated with human or horse serum), or a rabbit anti-C5b-9 polyclonal IgG (for spirochetes incubated with mouse serum) followed by an Alexa 647-conjugated goat anti-guinea pig IgG or goat anti-mouse IgG, or goat anti-rabbit IgG prior to being applied to flow cytometry analysis. Representative histograms of flow cytometry analysis showing the deposition levels of mouse **(A)** C3b or **(C)** MAC on the surface of indicated *B*. *burgdorferi* strains. The deposition levels of **(B)** C3b or **(D)** MAC of indicated animals on the surface of *B*. *burgdorferi* were measured by flow cytometry and presented as mean fluorescence index (MFI). Each bar represents the mean of three independent determinations ± SEM. Significant differences (P < 0.05 by one-way ANOVA with post hoc Bonferroni correction) in the deposition levels of C3b or MAC relative to the B313/Vector (“*”) or between two strains relative to each other (“#”).

We next aimed to determine the role of host-specific FH-binding of CspA variants in inhibiting complement deposition on the spirochete surface. We first incubated human, mouse, or horse serum with strain 5A4NP1Δ*cspA*-V or this strain producing CspA_B31_, CspA_PKo_, CspA_ZQ1_, or CspA_B31_L246D as well as the WT strain B31-5A15 or the negative control strain B313. The levels of C3 fragments (C3b and iC3b) and the MAC bound by spirochetes were quantified using flow cytometry. Quail serum was not included as antibodies against quail C3b or MAC were not available. FH-depleted serum was not used as the lack of FH in serum causes an uncontrollable complement activation, which consumes C3 resulting in no C3b/iC3b deposition on spirochete surface [41]. Consistent with previous observations [42], a significant amount of C3b and MAC was detected on the surface of the strain B313 carrying vector alone upon incubation with human (Fig 3B and 3D top panel), mouse, or horse serum (Fig 3B and 3D middle and bottom panels). Conversely, incubation of the WT strain B31-5A15 with serum from these animals resulted in at least 2-fold reduction of C3b deposition compared to strain B313, and virtually undetectable MAC deposition (Fig 3B and 3D, p < 0.05). Strain 5A4NP1Δ*cspA*-V had similar levels of C3b and MAC deposition compared to strain B313 (Fig 3B and 3D p > 0.05)[28]. These results indicate that CspA is required to inhibit human and non-human complement bound to the spirochete surface.

We also observed a correlation between the origin of the serum and the ability of CspA variants to inhibit deposition of C3 fragment or MAC: (1) Compared to strain 5A4NP1Δ*cspA*-V, expression of *cspA*_*B31*_ significantly decreased levels of C3b and MAC deposition on the spirochete surface in human serum (Fig 3B and 3D top panels, consistent with a previous finding [25]) and in mouse or horse sera (Fig 3A and 3C, the middle and bottom panels of Fig 3B and 3D). (2) Expression of *cspA*_*PKo*_ in the strain 5A4NP1Δ*cspA* significantly reduced human and mouse C3b and MAC deposition compared to strain 5A4NP1Δ*cspA*-V (Fig 3A and 3C, top and middle panels of Fig 3B and 3D), in agreement with a previous observation [25], but not in horse serum (bottom panels of Fig 3B and 3D). (3) Expression of *cspA*_*ZQ1*_ resulted in similar C3b and MAC deposition as the strain 5A4NP1Δ*cspA*-V in all three sera (Fig 3A to 3D). We also compared the complement activating abilities of isogenic 5A4NP1Δ*cspA* producing either CspA_B31_ or the FH-binding deficient point mutant CspA_B31_L246D. Although CspA_B31_ has also been shown to bind complement C7 and C9, and plasminogen [24, 25, 43], CspA_B31_L246D was selectively defective in FH-binding, but still bound to C7, C9, or plasminogen at levels similar to CspA_B31_ (S1 Table and S6 Fig). Thus, the CspA_B31_L246D producing strain showed significantly greater levels of C3 and MAC deposition than the strain producing CspA_B31_ (Fig 3B and 3D), suggesting that the FH-binding activity of CspA mediates inhibition of complement deposition on the surface of *B*. *burgdorferi*.

### CspA-mediated FH-binding activity facilitates bacterial serum survival in a host-specific fashion

**Fig 4:**
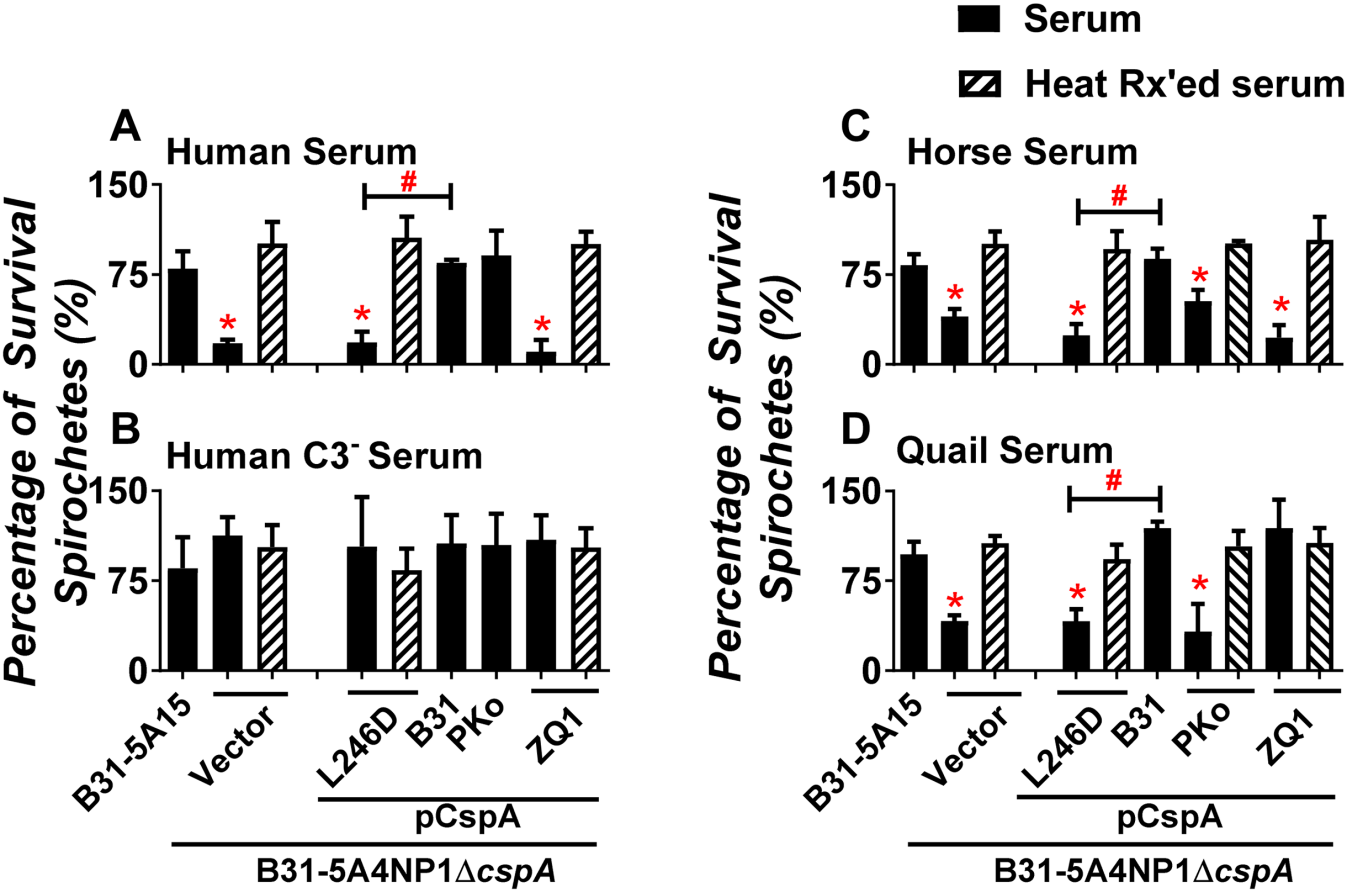
CspA variants mediate distinct levels of spirochete survival in serum from different vertebrate animals. *B*. *burgdorferi* strain B31-5A15 (“B31-5A15”), B31-5A4NP1Δ*cspA* harboring the vector pBSV2G (“Δ*cspA*/Vector”), or this *cspA* mutant strain producing CspA_B31_ (“Δ*cspA*/pCspA_B31_”), CspA_PKo_ (“Δ*cspA*/pCspA_PKo_”), CspA_ZQ1_ (“Δ*cspA*/pCspA_ZQ1_”), or CspA_B31_L246D (“Δ*cspA*/pCspA_B31_L246D”) was incubated for four hours with untreated (filled bars) or heat-inactivated (HI, hatched bars) serum with a final concentration of 40%. These sera include **(A)** human serum or **(B)** C3-depleted human serum (Human C3^-^ serum) or the serum from **(C)** horse or **(D)** quail. The number of motile spirochete was assessed microscopically. The percentage of survival for those *B*. *burgdorferi* strains was calculated using the number of mobile spirochetes at four hours post incubation normalized to that prior to the incubation with serum. Each bar represents the mean of three independent determinations ± SEM. Significant differences (P < 0.05 by one-way ANOVA with post hoc Bonferroni correction) in the percentage survival of spirochetes relative to the Δ*cspA*/Vector (“*”) or between two strains relative to each other (“#”).

The ability of pathogens to inhibit the host complement in the bloodstream correlates with their ability to survive in the serum [7]. Thus, we sought to investigate how species-to-species variation of CspA promotes bacterial survival in serum from different vertebrate hosts. We incubated WT strain B31-5A15, as well as strain 5A4NP1Δ*cspA*-V or this plasmid encoding *cspA*_*B31*_, *cspA*_*PKo*_, *cspA*_*ZQ1*_, or *cspA*_*B31*_*L246D* with serum from human, horse, or quail and negative control serum (C3-depleted or heat treated human serum) for four hours. Mouse serum was not used, as its complement is highly labile *ex vivo* [44], and the ability to kill spirochetes *in vitro* has not been consistently observed [45, 46]. More than 75% of the strain B31-5A15 survived in human serum or serum without active complement (C3-depleted serum) (Fig 4A and 4B). In addition, this strain survived in horse and quail serum, with average spirochete serum survival above 75% (Fig 4C and 4D). Less than 20% of the strain 5A4NP1Δ*cspA*-V remained motile after incubation with human serum (less than the survival percentage of WT strain B31-5A15, p < 0.05), but more than 85% of the strain 5A4NP1Δ*cspA*-V survived in the C3-depleted or heat inactivated human serum (Fig 4A and 4B). Similarly, 5A4NP1Δ*cspA*-V was killed by horse and quail serum, indicating that CspA plays an essential role in promoting spirochete survival in not only human serum as shown previously [28], but also in horse and quail serum (Fig 4C and 4D).

We also found that CspA variants produced on the strain 5A4NP1Δ*cspA* confer serum survival in a host-specific manner: (1) More than 75% of motile *cspA*_*B31*_-complemented *B*. *burgdorferi* were detected when incubated with sera from all these animals (Fig 4A, 4C and 4D). (2) A *cspA*_*PKo*_-complemented strain was able to survive in human serum (Fig 4A), but less than 50% of the spirochetes survived when incubated with horse or quail sera (Fig 4C and 4D). (3) Less than 20% of a *cspA*_*ZQ1*_-complemented strain survived following the treatment with human or horse serum (Fig 4A and 4C), but over 75% of this strain survived in quail serum (Fig 4D). Additionally, less than 25% of *cspA*_*B31*_*L246D*-complemented strain remained motile after treatments with human, horse, or quail serum, which was about three-fold lower than the *cspA*_*B31*_-complemented strain (Fig 4A, 4C and 4D). Conversely, more than 75% of *cspA*_*B31*_*L246D*-complemented strain survived in either C3-depleted or heat treated-serum, suggesting that the FH-binding ability of CspA promotes spirochete survival in serum (Fig 4A to 4D).

### *B*. *burgdorferi* CspA is up-regulated in post-molting but down-regulated in post-feeding nymphal ticks

**Fig 5:**
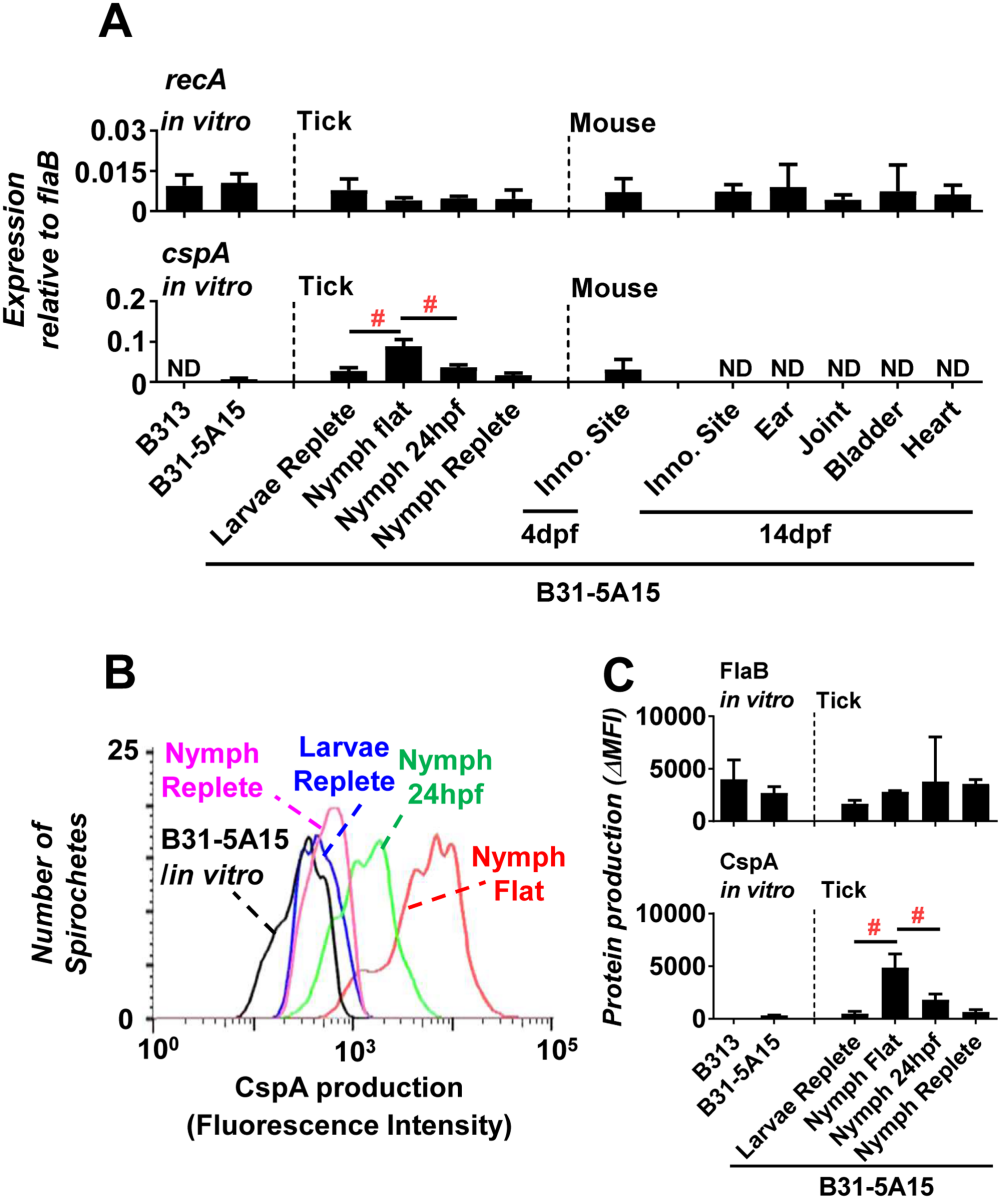
*B*. *burgdorferi* expresses distinct levels of CspA at different stages of enzootic cycle. C3H/HeN mice were infected with 10^5^ *B*. *burgdorferi* strain B31-5A15. At 14 days post infection, the uninfected *I*. *scapularis* larval ticks were allowed to feed on these mice to repletion. After the replete larvae molted into nymphs, these *B*. *burgdorferi*-infected nymphs were allowed to feed on naïve C3H/HeN mice for different period of time or to repletion. The mice were euthanized at 7 or 14 days post feeding (“7dpf” or “14dpf”) to collect the inoculation site of skin (for mice at 7 and 14 days post feeding), ears, tibiotarsus joints, bladder, and heart (for mice at 14 days post feeding). RNA was extracted from replete larvae (“larvae replete”), flat nymphs (“nymph flat”), fed nymphs at 24 hours post feeding (“24hpf”) or replete nymphs (“nymph replete”) as well as *in vitro* cultured *B*. *burgdorferi* strain B313 (“B313”, negative control) or B31-5A15 (“B31-5A15”, positive control) in BSKII medium. **(A)** RNA was also extracted from mouse tissues including tick biting site (“inoc. site”), ears, tibiotarsus joints, bladder, and heart at 7 and/or 14 days post feeding. The extracted RNA was then used to determine the expression levels of *cspA* and constitutive expressed genes *flaB* and *recA* using qRT-PCR (see Materials and methods). The expression levels of **(Top panel**; negative control) *recA* and **(Bottom panel)** *cspA* are presented by normalizing the expression levels of *flaB* (negative control) (see Materials and methods). Each bar represents the mean of five independent determinations ± SEM (“#”). Significant differences (P < 0.05 by one-way ANOVA with post hoc Bonferroni correction) in the normalized expression levels of *cspA* between two conditions relative to each other. **(B and C)** The bacteria isolated from ticks or *in vitro* cultured *B*. *burgdorferi* strains were applied to flow cytometry. **(B)** Representative histograms of flow cytometry analysis showing the production levels of CspA in *B*. *burgdorferi* in the indicated environment. **(C)** The production of FlaB (**Top panel**; negative control) and CspA **(Bottom panel)** are presented as “ΔMFI”, the mean fluorescence index obtained from each of these strains subtracting that obtained from the strains stained only by the secondary antibody. Each bar represents the mean of three independent determinations ± SEM. Significant differences (P < 0.05 by one-way ANOVA with post hoc Bonferroni correction) in the production of CspA relative to the B313 cultured *in vitro* (“*”) or between two conditions relative to each other (“#”).

We and others have demonstrated the binding of FH to CspA *in vitro* and the role of CspA in inactivating complement on spirochete surface and serum survival [25, 28, 30, 31] (Figs 2 to 4). How CspA promotes Lyme infection *in vivo* is still unclear. Thus, we examined regulation of CspA throughout the enzootic cycle (S7B Fig). The naïve larval *Ixodes scapularis* ticks were first allowed to feed on mice previously infected with the infectious *B*. *burgdorferi* strain B31-5A15. After the engorged and infected larval ticks molted into nymphal ticks, they were placed on naïve C3H/HeN mice for blood feeding. We then determined the expression levels of spirochete genes *cspA*, *recA*, and *flaB* in ticks and mouse tissues using quantitative RT-PCR and calculated the expression levels of *cspA* and *recA* relative to that of *flaB*. *recA* expression levels relative to *flaB* levels remained unchanged throughout the enzootic cycle (Fig 5A top panel). As reported previously [26], *cspA* expression was detected in both larval and nymphal ticks, as well as at tick bite sites on the mouse skin at 72 hours post feeding, but not in mouse tissues after spirochetes disseminate (Fig 5A bottom panel). *B*. *burgdorferi* in flat nymphs expressed more than 2-fold increased levels of *cspA* compared to levels prior to molting (replete larvae, p < 0.05), but the spirochetes’ *cspA* expression was reduced approximately 2-fold in nymphs following 24 hours of feeding, compared to expression levels in flat nymphs (Fig 5A bottom panel, p < 0.05).

We next sought to investigate CspA production in different stages of ticks. A previous study reported that the production of CspA could not be visually detected in spirochetes using fluorescence microscopy when spirochetes are present in nymphs [47]. One possibility for this discrepancy is that visual detection using microscopy may be subjective and not be sensitive enough to detect small differences in protein production [26]. We therefore gently lysed different stages of ticks, sorted *B*. *burgdorferi* from the tick lysates by their granularity and size, and quantitated the levels of CspA (and a constitutively produced protein FlaB) in the spirochetes using flow cytometry (S8 Fig). The strain B313, which does not carry the plasmid encoding *cspA*, was included as a negative control. There was no significant difference of FlaB production by strain B31-5A15 and the negative control strain B313 cultured *in vitro* (Fig 5C top panel). Additionally, we did not observe a significant difference of FlaB production by strain B31-5A15 in different stages of ticks (Fig 5C top panel). In spirochetes cultured *in vitro*, CspA production was detected in B31-5A15, but not in the strain B313 (Fig 5C bottom panel). Interestingly, we were able to detect *B*. *burgdorferi* CspA production in all examined stages of the ticks (significantly greater MFI values than strain B313; p < 0.05) (Fig 5C bottom panel). Further, the CspA production more than quadrupled in flat nymphs compared to fed larvae (p < 0.05), but thereafter halved after nymphs had fed for 24 hours (Fig 5B and 5C bottom panel). Our findings on the spirochetes’ CspA protein production in ticks are consistent with the dynamic changes of *cspA* mRNA levels (Fig 5A)[26], suggesting that CspA is up-regulated after larval ticks molt but is down-regulated after nymphal ticks feed.

### The *in vitro* FH-binding activity of CspA is correlated with spirochete survival in nymphs and transmission to mice

We then examined the role of CspA to facilitate spirochete survival in the enzootic cycle. As expected, when we inoculated C3H/HeN mice by subcutaneous needle infection with strain 5A4NP1Δ*cspA*-V or the WT strain B31-5A15 (S7A Fig), these strains colonized tissues to similar degrees (S9 Fig, 14 days post infection). Similarly, at early stages of infection, strain 5A4NP1Δ*cspA*-V colonized the skin of the mouse inoculation site and triggered bacteremia with a burden similar to the WT strain B31-5A15 (S10 Fig, 4 days post infection). These results indicate that CspA is not essential during early and disseminated stages of mouse infection through needle inoculation.

**Fig 6:**
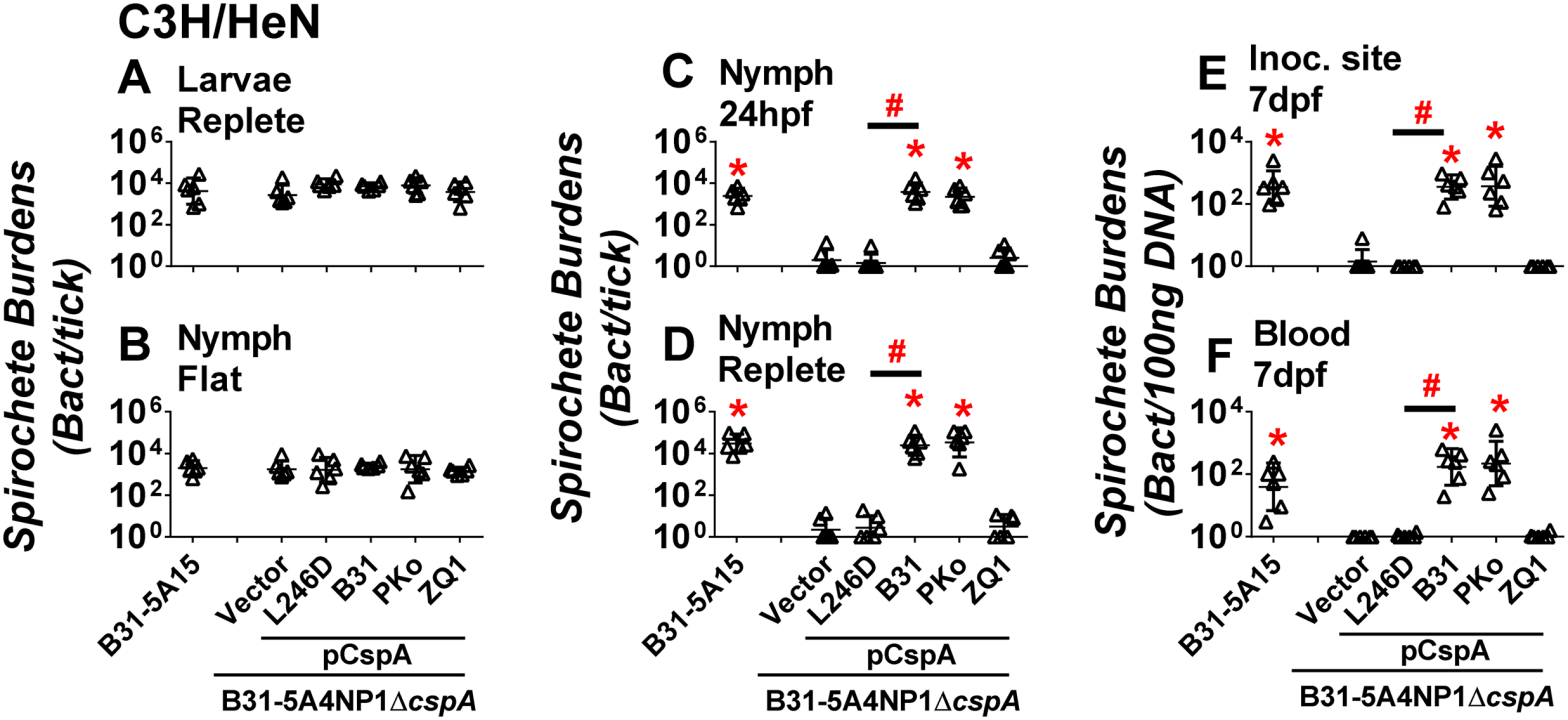
FH-binding ability of CspA promotes spirochete survival in nymphal ticks upon feeding. C3H/HeN mice were infected with 10^5^ *B*. *burgdorferi* strain B31-5A15 (“B31-5A15”), B31-5A4NP1Δ*cspA* harboring the vector pBSV2G (“Δ*cspA*/Vector”), or this *cspA* mutant strain producing CspA_B31_ (“Δ*cspA*/pCspA_B31_”), CspA_PKo_ (“Δ*cspA*/pCspA_PKo_”), CspA_ZQ1_ (“Δ*cspA*/pCspA_ZQ1_”), or CspA_B31_L246D (“Δ*cspA*/pCspA_B31_L246D”). At 14 days post infection, the uninfected *I*. *scapularis* larval ticks were allowed to feed on each of these mice until they are replete. After the replete larvae molt into nymphs, those *B*. *burgdorferi*-infected nymphs were allowed to feed on naïve C3H/HeN mice to repletion. The bacterial loads in the **(A)** replete larvae (“larvae replete”), **(B)** flat nymphs (“nymph flat”), fed nymphs at (**C)** 24 hours post feeding (“nymph 24hpf”) or **(D)** replete nymphs (“nymph replete”), or **(E)** the site where nymphal ticks fed (“inoc. site”) or **(F)** blood at 7 days post nymph feeding (“blood 7dpf”) were determined by qPCR. The bacterial loads in mouse tissues or blood were normalized to 100 ng total DNA. Shown are the geometric mean of bacterial loads ± 95% confidence interval of six ticks or mice per group. Significant differences (P < 0.05 by one-way ANOVA with post hoc Bonferroni correction) in the spirochete burdens relative to the Δ*cspA*/Vector (“*”) or between two strains relative to each other (“#”).

WT strain B31-5A15 and the strain 5A4NP1Δ*cspA*-V display similar levels of infectivity at 14 days post needle infection in mice (S9 Fig). Thus, to examine whether CspA plays a role *in vivo* during tick infection, we allowed the larval ticks to feed until replete on mice previously infected for 14 days with either WT strain B31-5A15 or strain 5A4NP1Δ*cspA*-V (S7B Fig). Nymphal ticks that developed after replete larval ticks molted were fed on naïve C3H/HeN mice until removed or replete, and the bacterial burdens in the nymphs and nymph-infected mouse blood and tissues were determined (S7B Fig). The bacterial loads of the strain 5A4NP1Δ*cspA*-V did not differ from the WT strain B31-5A15 in fed larvae or flat nymphs (Fig 6A and 6B). However, strain 5A4NP1Δ*cspA*-V did not survive in nymphs fed for 24, 48, or 72 hours or in replete nymphs (approximately 1000-fold less than the WT strain, Fig 6C and 6D and S11 Fig). Further, this strain was incapable of surviving in the mouse blood and colonizing tissues at 7 and 14 days post nymph feeding (at least 40-fold less than the WT strain, Fig 6E and 6F and S12 Fig). These results indicate that CspA enables the spirochete to survive in fed nymphs, which subsequently permits spirochete transmission to mice.

We then investigated the ability of CspA variants to promote spirochete survival in infected nymphs and in mice infected by tick infection. Mice were initially infected by needle injection with the strain 5A4NP1Δ*cspA* producing CspA_B31_, CspA_ZQ1_, or CspA_PKo_. All strains exhibited similar levels of tissue colonization (S9 Fig). After the larval ticks fed on these mice and molted into nymphs, the infected nymphs were placed on naïve mice (S7B Fig). All strains displayed similar levels of survival in fed larvae and flat nymphs (Fig 6A and 6B). The isogenic strain producing CspA_B31_ or CspA_Pko_ was able to survive in fed nymphs, colonize the mouse inoculation site, and survive in blood at the levels at least 150-fold more than the strain 5A4NP1Δ*cspA*-V (P < 0.05) (Fig 6C to 6F). Conversely (and similar to 5A4NP1Δ*cspA*-V), the isogenic strain producing CspA_ZQ1_ did not survive in nymphs fed for 24 hours or in replete nymphs and was incapable of surviving in the mouse blood and colonizing tissues (p > 0.05, Fig 6C to 6F). We then compared the burdens of the isogenic *B*. *burgdorferi* strain producing CspA_B31_ or the point mutant CspA_B31_L246D at different stages of tick and mouse infection. Similar levels of both strains were seen in fed larvae as well as in flat nymphs (Fig 6A and 6B). Interestingly, no CspA_B31_L246D-producing isogenic spirochetes were detectable in fed nymphs or at mouse inoculation site and in the bloodstream (similar strain 5A4NP1Δ*cspA*-V, p >0.05) (Fig 6C to 6F). These findings suggest that the *in vitro* FH-binding ability of CspA is correlated with spirochete survival in fed nymphs and with subsequent transmission to the mammalian host.

### FH binding to CspA enables complement evasion to permit spirochete survival in fed nymphal ticks and transmission to mammalian hosts

**Fig 7:**
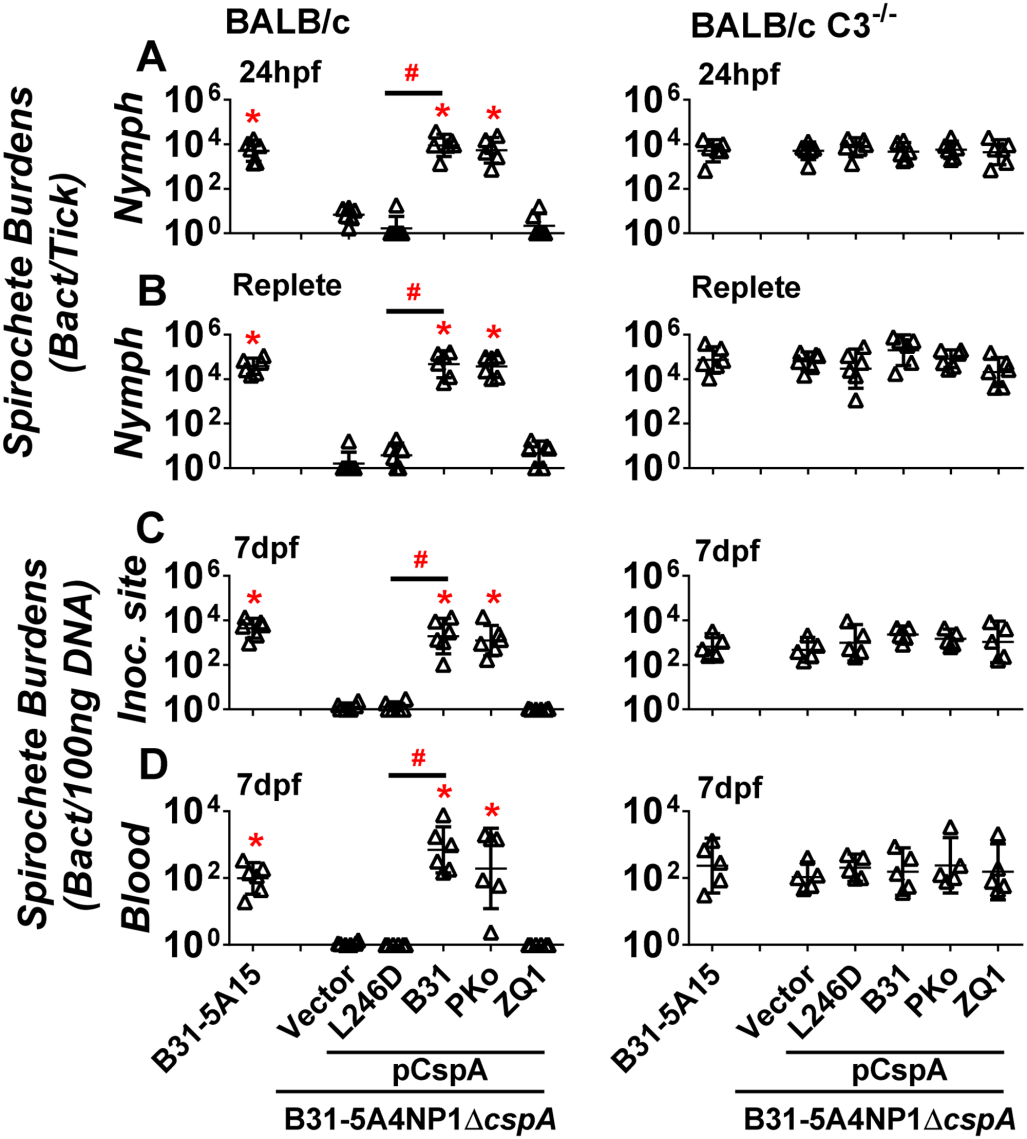
CspA-mediated FH-binding activity facilitates spirochete evading complement present in fed nymphs resulting in nymph-to-mouse transmission. C3H/HeN mice were infected with 10^5^ *B*. *burgdorferi* strain B31-5A15 (“B31-5A15”), B31-5A4NP1Δ*cspA* harboring the vector pBSV2G (“Δ*cspA*/Vector”), or this *cspA* mutant strain producing CspA_B31_ (“Δ*cspA*/pCspA_B31_”), CspA_PKo_ (“Δ*cspA*/pCspA_PKo_”), CspA_ZQ1_ (“Δ*cspA*/pCspA_ZQ1_”), or CspA_B31_L246D (“Δ*cspA*/pCspA_B31_L246D”). At 14 days post infection, the uninfected *I*. *scapularis* larval ticks were allowed to feed on each of these mice until they are replete. After the replete larvae molt into nymphs, those *B*. *burgdorferi*-infected nymphs were allowed to feed on naïve BALB/c or BALB/c C3^-/-^ mice to repletion. The bacterial loads in the nymphs fed on BALB/c **(Left panel)** or BALB/c C3^-/-^ **(Right panel)** for **(A)** 24 hours post feeding (“24hpf”) or **(B)** replete nymphs (“replete”), or at **(C)** the site where nymphs fed (“inoc. site”) on mice or **(D)** mouse blood at 7 days post nymph feeding (“7dpf”) were determined by qPCR. The bacterial loads in mouse tissues or blood were normalized to 100 ng total DNA. Shown are the geometric mean of bacterial loads ± 95% confidence interval of six ticks or mice per group. Significant differences (P < 0.05 by one-way ANOVA with post hoc Bonferroni correction) in the spirochete burdens relative to the Δ*cspA*/Vector (“*”) or between two strains relative to each other (“#”).

We have demonstrated that CspA is critical to promote spirochete survival in nymphal ticks in the first 24 hours of feeding (Fig 6). At this time point, the small amount of blood and the interstitial fluid that contain complement components enter nymphs’ midguts [48, 49]. The fact that the majority of *B*. *burgdorferi* have been found in the tick midgut at this time point (and is thus in contact with the host blood and interstitial fluid) [48, 50] led us to hypothesize that CspA-mediated complement inactivation facilitates host complement evasion by spirochetes in fed nymphs. Thus, we fed the nymphs infected with the WT strain B31-5A15 or the strain 5A4NP1Δ*cspA*-V on a mouse strain deficient in C3, the central molecule of complement required for opsonophagocytosis and formation of MAC (S7C Fig) [51]. WT BALB/c mice, which the C3-deficient mice were back-crossed into, served as controls. The spirochete burdens in the nymphs prior to and post feeding on mice were determined using qPCR. Consistent with infection in C3H/HeN mice, strain 5A4NP1Δ*cspA*-V was undetectable in the nymphs fed on WT BALB/c mice (Fig 7A and 7B, left panel) and at the bite site of the mouse skin, or in the blood (Fig 7C and 7D, left panel). However, this strain survived in nymphs fed on C3-deficient mice for 24 hours or to repletion at levels indistinguishable from the WT strain B31-5A15 (p > 0.05, Fig 7A and 7B, right panel). Strain 5A4NP1Δ*cspA*-V was also observed at bite sites and in the blood of C3-deficient mice at 7 days post nymph feeding (Fig 7C and 7D, right panel). These results suggest that CspA plays an essential role in evading the complement present in fed nymphs and for tick-to-mammal transmission.

We then examined the ability of *B*. *burgdorferi* strain B31-5A4NP1Δ*cspA* complemented with *cspA*_*B31*_, *cspA*_*ZQ1*_, or *cspA*_*PKo*_ to survive in nymphs fed on WT BALB/c or C3-deficient mice. In agreement with findings using WT C3H/HeN mice, the *cspA*_*B31*_- or *cspA*_*PKo*_- but not *cspA*_*ZQ1*_-complemented *B*. *burgdorferi* was detectable in the nymphs fed on WT BALB/c mice (Fig 7A and 7B, left panel) and at the bite sites of the skin and in blood of these mice (Fig 7C and 7D, left panel). Interestingly, the strain 5A4NP1Δ*cspA*-complemented with each of these three *cspA* alleles exhibited nearly identical bacterial levels in nymphs fed on C3-deficient mice for 24 hours or to repletion (Fig 7A and 7B, right panel). These *cspA*-complemented strains also colonized the tick bite sites and bloodstream at 7 days after the onset of nymph feeding at levels similar to WT bacteria (Fig 7C and 7D, right panel). These results suggest that the CspA variants differentially promote spirochete complement evasion in fed nymphs, which is required for spirochete transmission from ticks to mammals.

Nymphs infected with the *cspA*_*B31*_- or *cspA*_*B31*_*-L246D*-complemented strain were also allowed to feed on WT or C3-deficient BALB/c mice to test how FH binding by CspA promotes complement evasion in fed nymphs and facilitates tick-to-mammal transmission_._ We observed that the *cspA*_*B31*_*L246D*-complemented strain displayed approximately 5000-fold lower burdens than the *cspA*_*B31*_-complemented strain in nymphs fed on BALB/c mice for 24 hours or to repletion (Fig 7A and 7B, left panel). Similar to C3H/HeN mice, the *cspA*_*B31*_*L246D* strain was not detectable at the bite site or blood of BALB/c mice (Fig 7C and 7D, left panel). However, this mutant strain was detected in nymphs fed on C3-deficient mice for 24 hours, replete nymphs (Fig 7A and 7B, right panel), and at the bite sites and blood of C3 deficient mice at 7 days post feeding at levels similar to the *cspA*_*B31*_-complemented strain (Fig 7C and 7D, right panel). Our findings thus strongly suggest that the *in vitro* FH-binding activity of CspA is correlated with the ability of *B*. *burgdorferi* to evade mouse complement in fed nymphs, which would promote spirochete survival in those ticks and facilitate transmission from ticks to mammalian hosts.

## Discussion

Each of the Lyme borreliae species has been associated with specific vertebrate host(s) [7, 52, 53]. This spirochete-host association has been correlated with the ability of *B*. *burgdorferi* to survive in the blood (or serum) from the corresponding hosts [7, 54–56], but the mechanism that drives this association is still unclear. An attractive hypothesis is that the spirochetes exhibit host-specific immune evasion, which leads to the observed spirochete-host association. This could be due to variable spirochete outer surface proteins that interact with components of host complement in a host-specific manner. One such protein is CspA, which displays variant-to-variant differences in binding to human FH [25]. However, the recombinant CspA protein from *B*. *burgdorferi* strain B31 is incapable of binding to any other vertebrate animals’ FH, when the FH has been subjected to SDS-PAGE followed by a far-western blot [57]. In contrast, FH from the serum of multiple animals including human and mouse recognizes CspA variants run on a similar blot [58, 59]. This discrepancy may be due to structural alternations of animals’ FH on SDS-PAGE and the following far-western blot [8, 22, 23]. Therefore, we further verified the binding of CspA to FH from mouse, horse, and quail by demonstrating that the CspA of three main Lyme borreliae (*B*. *burgdorferi*, *B*. *afzelii*, or *B*. *garinii*) bind to purified FH in a host-specific manner. Additionally, we observed that the ability of different CspA variants to bind to FH correlates with their ability to inhibit complement activation on the spirochete surface and facilitate spirochete survival in serum in a host-specific manner. We found that these correlations not only apply to human complement (consistent with a previous study [28]) but also to complement of other animals. Further, the high-resolution structure of CspA suggests the recombinant version of this protein forms a dimer [35]. Two FH-binding regions have been localized on the central cleft of the CspA dimer and on the C-termini of this protein including leucine-246 [34, 60]. By ectopically producing a CspA point mutant (CspA-L246D) selectively lacking FH-binding ability on spirochetes, we further demonstrated that the CspA-mediated FH-binding activity contributes to the inactivation of host complement and spirochete survival in sera. Note that similar non-polar features of this position (leucine-246) of CspA_B31_ and the equivalent location of other CspA variants (phenylalanine-237 of CspA_PKo_ and leucine-252 of CspA_ZQ1_) suggests a possibility that this amino acid is critical for the FH-binding activity of these variants (S1 Fig).

FH is polymorphic between humans and mice (61% amino acid identity) [13, 14]. However, we have demonstrated a similar ability of CspA to bind to both human and mouse FH as well as inhibit complement deposition of both human and mouse sera on the spirochete surface. These findings imply that CspA plays a similar role in mouse and human during infections. Thus, the murine model was selected to test the role of the CspA-mediated FH binding activity. In addition, we have demonstrated that spirochetes require CspA to survive in fed nymphs during tick-to-mouse transmission. This result is consistent with the fact that *cspA* is uniquely expressed when spirochetes are in fed nymphs [26]. In contrast, CspA was not essential for spirochetes to be transferred from mouse to larvae or establish infection in mouse skin at early stages of infection, even though *cspA* expression was detectable at these stages. This may be due to the previous observation that other genes encoding functionally redundant FH-binding proteins (e.g. *cspZ*, *erpP*, and *erpA*) are co-expressed with *cspA* in spirochetes at these stages [26]. Unexpectedly, CspA was detected in spirochetes when spirochetes are in flat nymphs even though this protein was not required for *B*. *burgdorferi* to survive in these ticks. One possibility is that spirochetes may need to maintain certain levels of CspA in preparation to survive in the first 24 hours of feeding when the small amount of blood and interstitial fluid containing complement components enter the ticks’ midgut. This possibility is supported by the observations that a *cspA* deficient *B*. *burgdorferi* is eliminated from human serum within 1 hour [28], but a significant shift in gene expression is not detectable until the spirochetes are treated with blood for 48 hours [61].

In addition to CspA, *B*. *burgdorferi* requires other proteins to promote persistent survival in fed nymphs and to be transmitted to vertebrate animals [62–66]. Some of these proteins have been thought to be important for nutrient acquisition and metabolism in ticks [62, 67–72], while the functions of the other proteins are still unclear. Blood and interstitial fluid from vertebrate hosts contain diverse innate immune defense mechanisms, including complement [9]. In fact, *B*. *burgdorferi* displays increased infectivity in C3-deficient mice, which lack the ability to deposit opsonic C3 fragments or to generate the pore-forming MAC that can lyse spirochetes [73, 74]. This finding suggests that spirochetes need to evade hosts’ complement to survive in vertebrate animals. Moreover, Rathinavelu et al. reported that the blood meals from either WT or C3-deficient mice do not eliminate the WT *B*. *burgdorferi* in fed ticks [75], raising the possibility that spirochetes produce factors to facilitate the complement evasion in fed ticks. In this study, we observed a clear correlation of CspA variants or mutants in their FH-binding activity with their ability to promote spirochete survival in nymphs fed on WT mice and tick-to-mouse transmission. Conversely, this correlation was not detectable when these ticks fed on C3-deficient mice. These findings identify that CspA-mediated FH-binding activity is necessary for the spirochetes’ evasion of complement in fed nymphs and eventually to be transmitted to mammalian hosts. It is noteworthy that Woodman et al. reported that FH-deficient mice were susceptible to WT *B*. *burgdorferi* infection, leading to the conclusion that spirochetes did not require FH-binding activity to evade mouse complement [74]. However, the lack of the FH leads to the spontaneous complement activation and subsequent complement consumption in these mice, rendering them functionally complement deficient [41]. Thus, FH-deficient mice cannot be used to study the role of FH-binding microbial proteins in complement evasion.

**Fig 8:**
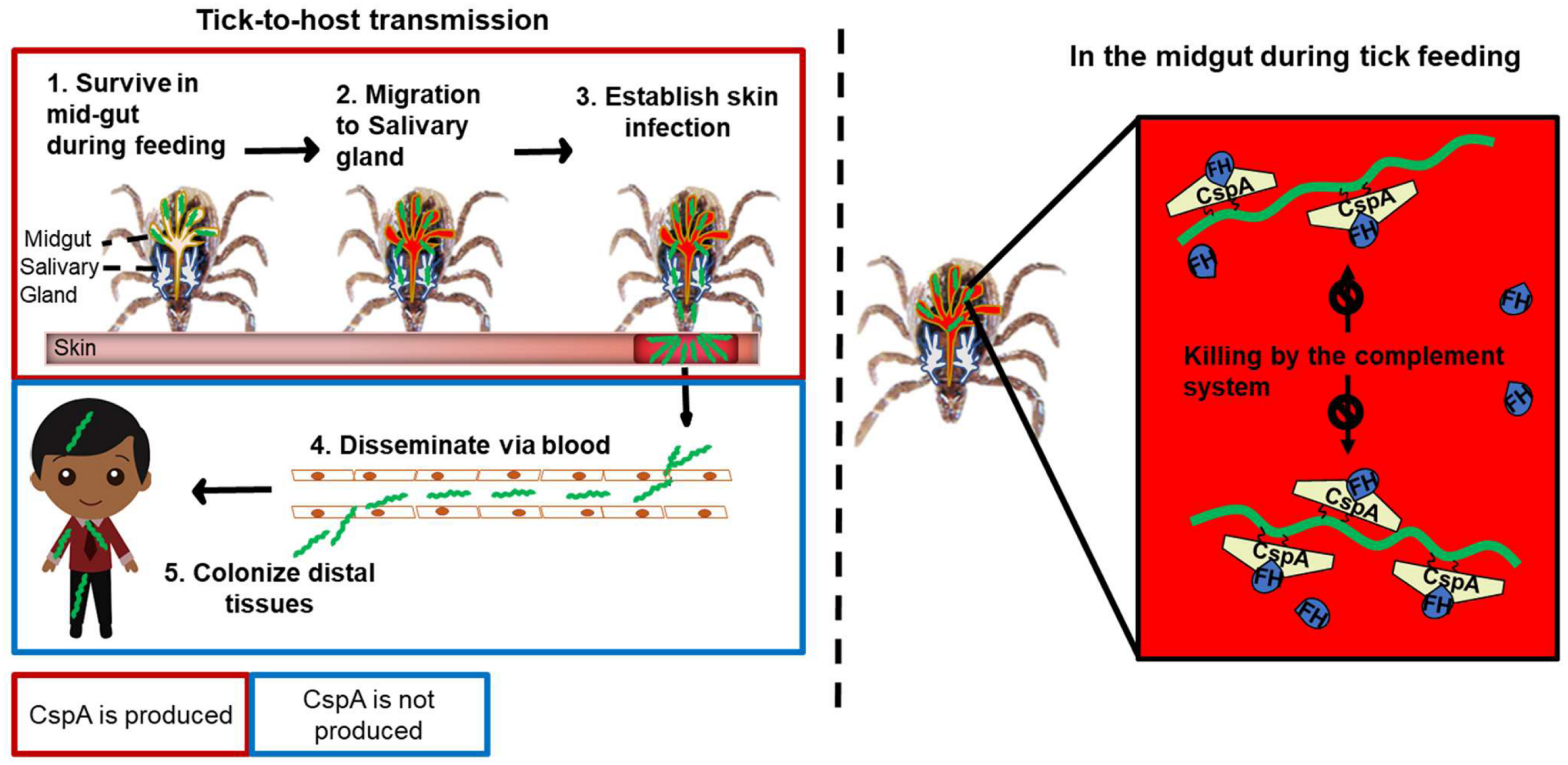
A proposed model showing CspA-mediated FH-binding activity promotes spirochete evasion of complement in feeding nymphs to facilitate tick-to-host transmission. CspA of spirochetes binds to FH present in the host’s blood and interstitial fluid in feeding nymphs to facilitate spirochete escape from the complement-mediated killing. Such CspA-mediated immune evasion ultimately facilitates spirochete transmission from nymphs to hosts.

It is noteworthy that *B*. *garinii* ZQ1 was originally isolated from ticks [76]. Therefore, the infectivity of this strain in vertebrate animals is still unclear. We found that variant to variant differences of CspA-mediated FH-binding activity are correlated with these variants’ ability to confer spirochete transmission from nymphs to mice. Particularly, the *cspA-ZQ1*-complemented strain infected C3-deficient mice but not wild type mice via ticks. Thus, our observation of CspA-ZQ1 selectively binding to quail FH implies that this variant may promote spirochete infectivity in quail by evading this animal’s complement. (Note that the complement among different avian hosts may vary. Thus, that one CspA variant promotes infectivity in quail may not necessarily indicate that the same variant contributes to the infectivity in other birds). More in-depth studies on avian hosts promoted by CspA-ZQ1 are warranted. In this study, we demonstrated the molecular mechanisms by which CspA of *B*. *burgdorferi* facilitates complement evasion of spirochetes in fed nymphal ticks (Fig 8). The host-specific differences in FH-binding capabilities conferred by CspA variants illuminate the possibility of a complement-driven host-specificity and selective transmission of Lyme disease spirochetes.

## Materials and methods

### Ethics statement

All mouse experiments were performed in strict accordance with all provisions of the Animal Welfare Act, the Guide for the Care and Use of Laboratory Animals, and the PHS Policy on Humane Care and Use of Laboratory Animals. The protocol was approved by the Institutional Animal Care and Use Committee (IACUC) of Wadsworth Center, New York State Department of Health (Protocol docket number 16–451), and University of Massachusetts Medical School (Protocol docket number 1930). All efforts were made to minimize animal suffering.

### Mouse, tick, and bacterial strains

C3H/HeN, BALB/c and Swiss-Webster mice were purchased from Charles River (Boston, MA) and Taconic (Hudson, NY), respectively. C3^-/-^ mice (C57BL/6) purchased from Jackson Laboratory (Bar Harbor, ME) were backcrossed for 11 generations into BALB/c background. Mice were genotyped for the C3 allele by PCR analysis of mouse tail DNA. *Ixodes scapularis* tick larvae were purchased from National Tick Research and Education Center, Oklahoma State University (Stillwater, OK) or obtained from BEI Resources (Manassas, VA). *B*. *burgdorferi*-infected nymphs were generated as described in the section “Mouse infection experiments by ticks.” The *Borrelia* and *Escherichia coli* strains used in this study are described in S2 Table. *E*. *coli* strains DH5α, M15, and derivatives were grown in Luria-Bertani (BD Bioscience, Franklin lakes, NJ) broth or agar, supplemented with kanamycin (50 μg/mL), ampicillin (100 μg/mL), or no antibiotics where appropriate. All *B*. *burgdorferi*, *B*. *afzelii*, and *B*. *garinii* strains were grown in BSK-II completed medium supplemented with kanamycin (200 μg/mL), streptomycin (50 μg/mL), gentamicin (50 μg/mL), or no antibiotics (see S2 Table).

### Generation of recombinant CspA proteins and antisera

The open reading frames lacking the putative signal sequences of *bba68* (*cspA*_*B31*_) from *B*. *burgdorferi* strains B31 or *zqa68* (*cspAZQ1*) from *B*. *garinii* strain ZQ1 were amplified using primers listed in S3 Table to generate recombinant histidine-tagged CspA proteins. In addition, an altered open reading frame encoding CspA_B31_L246D (residue 26 to 252 of CspA_B31_ with leucine-246 replaced by aspartate) was amplified using the primers described in S3 Table. Amplified fragments were engineered to encode a BamHI site at the 5’ end and a stop codon followed by a SalI site at the 3’ end. PCR products were sequentially digested with BamHI and SalI and then inserted into the BamHI and SalI sites of pQE30Xa (Qiagen, Valencia, CA). The plasmids were transformed into *E*. *coli* strain M15, and the plasmid inserts were sequenced (Wadsworth ATGC core facility). The resulting M15 derived strains and the M15 strain carrying the plasmid encoding the open reading frames lacking the putative signal sequences of *bafPKo_A0067* (*cspA*_*PKo*_) from *B*. *afzelii* strains PKo [25] were used to produce respective recombinant CspA variants or mutants (S2 Table). The histidine-tagged CspA variants or mutants were produced and purified by nickel affinity chromatography according to the manufacturer’s instructions (Qiagen, Valencia, CA). Antisera against CspA_B31_, CspA_PKo_, or CspA_ZQ1_ were generated by immunizing four-week-old Swiss Webster mice with each of the CspA proteins as described previously [77]. The ability of each of these antisera to recognize CspA_B31_, CspA_PKo_, CspA_ZQ1_, or CspA_B31_L246D was verified using ELISA. Basically, one microgram of the above-mentioned CspA variants or mutant proteins, or BSA (negative control) was immobilized on microtiter plates (MaxiSorp, ThermoFisher). The antisera raised from the mice immunized with each of these CspA variants (1: 1, 000x) were added to the wells. The pre-immune mouse serum was also included as a negative control. HRP-conjugated goat anti-mouse IgG (1: 1,000x) (ThermoFisher) was then added as antibody to detect the binding of the mouse anti-sera to CspA variants. The plates were washed three times with PBST (0.05% Tween 20 in PBS), and 100 μL of ortho-phenylenediamide dihydrochloride solution (Sigma-Aldrich) were added to each well and incubated for five minutes. The reaction was stopped by adding 50 μL of 2.6M hydrosulfuric acid to each well. Plates were read at 405nm using a Tecan Sunrise Microplate reader (Tecan, Morrisville NC). The anti-sera of CspA_B31_, CspA_PKo_, CspA_ZQ1_, or CspA_B31_L246D exhibited similar levels of binding to each of these CspA variants or mutant proteins (S13 Fig).

### Circular Dichroism (CD) spectroscopy

CD analysis was performed on a Jasco 810 spectropolarimeter (Jasco Analytical Instrument, Easton, MD) under nitrogen. CD spectra were measured at room temperature (RT, 25°C) in a 1 mm path length quartz cell. Spectra of CspA_B31_ (10 μM) or CspA_B31_L246D (10 μM) were recorded in phosphate based saline buffer (PBS) at RT, and three far-UV CD spectra were recorded from 190 to 250 nm for far-UV CD in 1 nm increments. The background spectrum of PBS without proteins was subtracted from the protein spectra. CD spectra were initially analyzed by the software Spectra Manager Program (Jasco). Analysis of spectra to extrapolate secondary structures and the prediction of the spectrum using the amino acid sequences of CspA_B31_ were performed by Dichroweb (http://dichroweb.cryst.bbk.ac.uk/html/process.shtml) using the K2D and Selcon 3 analysis programs [78].

### Purification of horse and quail FH

The procedure to purify FH from serum of various vertebrate animals has been described previously [79]. Basically, the serum collected from horses originally from New Zealand, (ThermoFisher, Waltham, MA) or *Coturnix coturnix* quail (Canola Live Poultry Market, Brooklyn, NY) was centrifuged to remove aggregates prior to being diluted with two volumes deionized water. Then, 6g of cyanogen bromide (CNBr)-activated Sepharose 4B resin (GE Healthcare, Piscataway, NJ) was mixed with 100mg of Trinitrophenyl-Bovine Serum Albumin (TNP-BSA) (LGC Biosearch Technology, Petaluma, CA) for 2 hours followed by incubation with the blocking buffer (PBS with 100mM ethanolamine-HCl, 150mM NaCl at pH8.5) at room temperature for 2 hours. The TNP-BSA CNBr resin was then equilibrated with PBS and packed into a column. After the diluted serum was applied into the TNP-BSA CNBr column, the column was washed by PBS until the OD_280_ values of the effluent below 0.04 Arbitrary Unit. Bound proteins were then eluted by the elution buffer (PBS with 0.5mM EDTA and 1M sodium chloride at pH7.4). The eluent was subsequently applied to a NAb Protein G Spin Column (ThermoFisher) according to the manufacturer’s instruction to remove the immunoglobulin in the serum. The purified factor H was confirmed by ELISA [77]. A sheep anti-FH polyclonal IgG (ThermoFisher) (1:200x), which has been shown to recognize horse FH [58] or a mouse anti-FH monoclonal antibody VIG8 (1: 200x), which has been observed to recognize avian FH [80] was used as a primary antibody. A horse radish peroxidase (HRP) conjugated donkey anti-sheep (1:2,000x) (ThermoFisher) or goat anti-mouse (1: 1,000x) was used as a secondary antibody.

### FH binding assay by quantitative ELISA

Quantitative ELISA for FH, C7, C9, or plasminogen binding by CspA proteins was performed similarly to that previously described [78]. For FH binding, one microgram of BSA (negative control; Sigma-Aldrich) or FH from human (ComTech, Tyler, Texas), mouse (MyBiosource, San Diego, CA)[81], horse, or quail was coated onto microtiter plate wells. For FH binding, one hundred microliters of increasing concentrations (0.03125, 0.0625, 0.125, 0.25, 0.5, 1, 2 μM) of histidine-tagged DbpA from *B*. *burgdorferi* strain B31 (negative control) or a CspA variant or mutant, including CspA_B31_, CspA_PKo_, CspA_ZQ1_, or CspA_B31_L246D was then added to the wells. Mouse anti-histidine tag (Sigma-Aldrich, St. Louis, MO; 1:200) and HRP-conjugated goat anti-mouse IgG (1: 1,000x) were used as primary and secondary antibodies, respectively, to detect the binding of histidine-tagged proteins. The plates were washed three times with PBST (0.05% Tween 20 in PBS), and 100 μL of tetramethyl benzidine (TMB) solution (ThermoFisher) were added to each well and incubated for five minutes. The reaction was stopped by adding 100 μL of 0.5% hydrosulfuric acid to each well. Plates were read at 405 nm using a Tecan Sunrise Microplate reader. To determine the dissociation constant (K_D_), the data were fitted by the following equation using GraphPad Prism software (Version 7, La Jolla, CA).

$$OD405=\frac{\text{OD}405\text{max}\left[\text{CspA}\;\text{proteins}\right]}{\text{KD}+\left[\text{CspA}\;\text{proteins}\right]}$$ (1)

### Surface plasmon resonance (SPR)

Interactions of CspA with FH were analyzed by a SPR technique using a Biacore 3000 (GE Healthcare). Ten micrograms of FH from human, mouse, or horse were conjugated to a CM5 chip (GE Healthcare) as described previously [78]. A control flow cell was injected with PBS without FH. For quantitative SPR experiments to determine FH-binding, ten microliters of increasing concentrations of CspA variants or mutants, including CspA_B31_, CspA_PKo_, CspA_ZQ1_, or CspA_B31_L246D, were injected into the control cell and flow cell immobilized with different animals’ FH, human plasminogen (Sigma-Aldrich), C7 (ComTech), or C9 (ComTech) at 10 μL/min, 25°C. To obtain the kinetic parameters of the interaction, sensogram data were fitted by means of BIAevaluation software version 3.0 (GE Healthcare), using the one step biomolecular association reaction model (1:1 Langmuir model), resulting in optimum mathematical fit with the lowest Chi-square values.

### Shuttle plasmid construction

*cspA*_*B31*_, *cspA*_*PKo*_, *cspA*_*ZQ1*_, or *cspA*_*B31*_*L246D* was first PCR amplified with the addition of a SalI site and a BamHI site at the 5’and 3’ ends, respectively, using Taq polymerase (Qiagen) and the primers (see S3 Table) to generate the plasmids encoding *cspA* alleles. The unpaired nucleotides at 5’ and 3’ end of the amplified DNA fragments were removed by exonuclease from CloneJet PCR cloning kit (ThermoFisher) and then inserted into the vector pJET1.2/blunt (ThermoFisher). The plasmids were then digested with SalI and BamHI to release the *cspA* alleles, which were then inserted into the SalI and BamHI sites of pBSV2G (S2 Table) [82]. The promoter region of *cspA* from *B*. *burgdorferi* B31, 400bp upstream from the start codon of *cspA*, was also PCR amplified. SphI and SalI sites were added at the 5’and 3’ ends of amplified DNA, respectively, using primers pcspAfp and pcspArp (S3 Table). Promoter fragments were then inserted into the SphI and SalI sites of pBSV2G to drive the expression of *cspA*_*B31*_, *cspA*_*PKo*_, *cspA*_*ZQ1*_, and *cspA*_*B31*_*L246D*.

### Plasmid transformation into *B*. *burgdorferi*

Electrocompetent *B*. *burgdorferi* B31-5A4NP1Δ*cspA* prepared as described [77, 83] was transformed separately with 80 μg of each of the shuttle plasmids encoding *cspA*_*B31*_, *cspA*_*PKo*_, *cspA*_*ZQ1*_, or *cspA*_*B31*_*L246D* (S2 Table) and cultured in BSK II medium at 33°C for 24 hours. Liquid plating transformations were performed in the presence of antibiotic selection (50 μg/mL gentamicin, 200μg/mL kanamycin, 50μg/mL streptomycin, as required), as described previously [84, 85]. After incubation at 33°C in 5% CO_2_ for two weeks, kanamycin-, gentamicin-, and streptomycin-resistant colonies of *cspA*-complemented *B*. *burgdorferi* were obtained and expanded at 33°C in liquid BSK II medium containing these antibiotics, followed by genomic DNA preparation as previously described [86]. PCR was performed with primers (S3 Table) specific for *aphI* (encoding the kanamycin resistance gene), *aacC1* (encoding the gentamicin resistance gene), and *aadA* (encoding the streptomycin resistance gene) to verify their presence in the transformants. The plasmid profiles of the *cspA* deficient mutant complemented with *cspA* alleles were examined as described previously [36]. The plasmid profiles of these strains were found to be identical to those of the parental strain 5A4NP1Δ*cspA* and the strain 5A4NP1Δ*cspA*-V.

### Surface localization of CspA detected by flow cytometry

The determination of surface localization of CspA by Flow cytometry has been described previously [77, 87, 88]. Basically, 1 x 10^8^ *B*. *burgdorferi* cells producing CspA_B31_, CspA_PKo_, CspA_ZQ1_, or CspA_B31_L246D were washed three times with HBSC buffer containing glucose and BSA (25 mM Hepes acid, 150 mM sodium chloride, 1 mM MnCl_2_, 1 mM MgCl_2_, 0.25 mM CaCl_2_, 0.1% glucose, and 0.2% BSA, final concentration) and then resuspended into 500 μL of the same buffer. To prepare permeabilized spirochetes, 1 × 10^8^ *B*. *burgdorferi* was incubated with 100% methanol for an hour, followed by washing three times with HBSC buffer containing glucose and BSA. A mixture of mouse antisera raised against CspA_B31_, CspA_PKo_, or CspA_ZQ1_ or monoclonal mouse antibody against *B*. *burgdorferi* FlaB (negative control) was used as a primary antibody (1:250x). An Alexa 647-conjugated goat anti-mouse IgG (ThermoFisher) (1:250x) was used as a secondary antibody. Three hundred microliters of formalin (0.1%) were then added for fixing. Surface production of CspA was measured by flow cytometry using a Becton-Dickinson FACSCalibur (BD Bioscience). All flow cytometry experiments were performed within two days of collection of *B*. *burgdorferi* samples. Spirochetes in the suspension were distinguished on the basis of their distinct light scattering properties in the flow cytometer equipped with a 15 mW, 488 nm air-cooled argon laser, a standard three-color filter arrangement, and CELLQuest Software (BD Bioscience). The mean fluorescence index (MFI) of each sample was obtained from FlowJo software (Three-star Inc, Ashland, OR) representing the surface production of the indicated proteins. Mean Fluorescence Index (MFI) normalized to that of CspA from permeabilized *B*. *burgdorferi* obtained from S5 Fig was used to compare the surface production of CspA in different strains. These results represent the mean of three independent determinations ± the standard deviation of mean. Each standard deviation of mean value was no more than 7% of its mean value.

### FH-binding assay of *B*. *burgdorferi* detected by flow cytometry

To quantitatively determine the ability of *B*. *burgdorferi* strains producing CspA_B31_, CspA_PKo_, CspA_ZQ1_, or CspA_B31_L246D in binding to FH, 1 x 10^7^ *B*. *burgdorferi* strains were washed twice by PBS, resuspended into 100μL of the same buffer, and then incubated with FH from human, mouse, horse, or quail (1 μg per reaction) or C3-depleted human serum (ComTech) or serum from BALB/c C3^-/-^ mice (Final concentration: 20%) at 25°C for 1 hour. Following incubation, the spirochetes were washed three times with PBS and resuspended in 100μL of HBSC buffer containing DB. A sheep anti-FH polyclonal IgG (ThermoFisher) (1:250x), which has been shown to recognize FH from human, mouse and horse [58] or a mouse anti-FH monoclonal antibody VIG8 (1:250x), which has been observed to recognize avian FH [80] were used as primary antibodies. An Alexa 647-conjugated donkey anti-sheep IgG (ThermoFisher) (1: 250x) or goat anti-mouse IgG (1:250x) was used as secondary antibodies. Three hundred microliters of formalin (0.1%) was then added for fixing. The mean fluorescence index (MFI) of each *B*. *burgdorferi* strain was measured to determine the FH-binding capability promoted by CspA variants or mutants using a Becton-Dickinson FACSCalibur and analyzed by FlowJo software as described above.

### C3b and MAC deposition assay on *B*. *burgdorferi* detected by flow cytometry

The deposition of C3b and MAC on the surface of *B*. *burgdorferi* producing CspA_B31_, CspA_PKo_, CspA_ZQ1_, or CspA_B31_L246D was quantitatively determined by flow cytometry as revised from previous reported methodologies [89]. *B*. *burgdorferi* strains were washed twice, resuspended in PBS, and then incubated with serum from human (MP Biomedical, Santa Ana, CA), mouse (Southern Biotech, Birmingham, AL), horse, or quail (Final concentration: 20%) at 25°C for 1 hour. Twenty percent serum was used in this study because more than 80% of *B*. *burgdorferi* strains were capable of surviving in this concentration of serum [28], but C3b and MAC have been constantly detected on spirochete surface when these strains are incubated with 20% serum [28]. Prior to being mixed with *B*. *burgdorferi*, those sera were screened with the C6 Lyme ELISA kit (Diamedix, Miami Lakes, FL) to determine whether the individual from which it was collected had prior exposure to *B*. *burgdorferi* by detecting the antibody against the C6 peptide of a *B*. *burgdorferi* protein VlsE [90]. Then, the spirochetes were washed three times with PBS and resuspended in HBSC buffer containing glucose and BSA. A guinea pig anti-C3 polyclonal IgG (ThermoFisher) (1:250x), which has been shown to recognize C3 from human, mouse and horse, was used as a primary antibody to detect C3b. A mouse anti-C5b-9 monoclonal antibody aE11 (ThermoFisher) (1:250x), which has been observed to recognize MAC from human and horse, and a rabbit anti-C5b-9 polyclonal IgG (Abcam, Cambridge, MA) (1:250x), which has been verified to bind to MAC from human and mouse, were used as primary antibodies. An Alexa 647-conjugated goat anti-guinea pig IgG (ThermoFisher) (1:250x), goat anti-mouse IgG (ThermoFisher) (1:250x), or goat anti-rabbit IgG (ThermoFisher) (1:250x) were used as secondary antibodies. Three hundred microliters of formalin (0.1%) were then added for fixing. The mean fluorescence index (MFI) of each *B*. *burgdorferi* strain was measured to determine the levels of C3b or MAC deposition on the surface of *B*. *burgdorferi* strains using a Becton-Dickinson FACSCalibur and analyzed by FlowJo software as described above.

### Serum sensitivity assay

The serum sensitivity of *B*. *burgdorferi* strain B31-5A15, B31-5A4NP1Δ*cspA*-V and this *cspA* mutant strain producing CspA_B31_, CspA_PKo_, CspA_ZQ1_, or CspA_B31_L246D was measured using a published procedure [91]. Briefly, triplicate samples of each strain were grown to mid-log phase and diluted to a final concentration of 5×10^6^ bacteria per milliliter into BSKII medium without rabbit serum with a final concentration of 40% normal serum from human, mouse, horse, or quail or C3-depleted human serum. We also included the spirochetes mixed with 40% heat-inactivated serum from these vertebrate hosts, which was incubated at 55 °C for 2 hours prior to being mixed with spirochetes. At 0 and 4 hours after the addition of serum, an aliquot was taken from each condition and counted by Petroff-Hausser counting chamber (Hausser Scientific, Horsham, PA) using a Nikon Eclipse E600 darkfield microscope (Nikon, Melville, NY). Though the strain B31-5A4NP1Δ*cspA* has been shown to be eliminated by incubating human serum (final concentration 40%) in one hour [28], the survival of the B31-5A4NP1Δ*cspA*-derived strains was evaluated at 4 hours post incubation to delineate the potential partial serum survival of these strains. The percentage of survival for those *B*. *burgdorferi* strains was calculated using the number of mobile spirochetes at 4 hours post incubation normalized to that prior to the incubation with serum.

### Mouse infection experiments using needle inoculation

Four-week-old female C3H/HeN mice were used for experiments involved in needle infection of *B*. *burgdorferi*. Mice were infected by intradermal injection as previously described [77] with 10^5^ of different strains of *B*. *burgdorferi* strain B31-5A15, B31-5A4NP1Δ*cspA*-V or this *cspA* mutant strain producing CspA_B31_, CspA_PKo_, CspA_ZQ1_, or CspA_B31_L246D. The plasmid profiles and the presence of the shuttle vector of each of these *B*. *burgdorferi* strains were verified prior to infection as described to ensure the stability of the vector and no loss of plasmids (S7A and S7B Fig) [36]. Mice were sacrificed at 14 days post-infection, the inoculation site of the skin, the tibiotarsus joints, ears, bladder, and heart were collected to quantitatively evaluate levels of colonization during infection.

### Mouse infection experiments by ticks

The procedure of the tick infection has been shown in S7B and S7C Fig and described previously [92]. Basically, four-week-old male and female C3H/HeN mice were infected with 10^5^ of *B*. *burgdorferi* strain B31-5A15, the *cspA* knockout mutant strain B31-5A4NP1Δ*cspA*-V or this *cspA* mutant strain producing CspA_B31_, CspA_PKo_, CspA_ZQ1_, or CspA_B31_L246D by intradermal injection as described above. The ear punches from those mice were collected and placed into BSKII medium at 7 days post infection, and the spirochete growth in the medium was evaluated to confirm the infection of these mice. At 14 days post infection, the uninfected larvae were allowed to feed to repletion on those *B*. *burgdorferi*-infected C3H/HeN mice as described previously [92]. Approximately 100 to 200 larvae were allowed to feed on each mouse. The engorged larvae were collected and allowed to molt into nymphs in 4 to 6 weeks in a desiccator at room temperature and 95% relative humidity in a room with light dark control (light to dark, 12: 12 hours). DNA was extracted from engorged larvae and post molting flat nymphs to examine the plasmid profiles and the presence of the shuttle vector the *B*. *burgdorferi* strains carried by these ticks as described to ensure no loss of plasmids during acquisition and molting (S7B Fig) [36]. The flat nymphs molted from larvae were placed in a chamber on four to six-week old male and female C3H/HeN, BALB/c, or C3^-/-^ mice in BALB/c background as described [93]. Ten nymphs were allowed to feed on each mouse. After the nymphs were forcibly removed by forceps at 24, 48, or 72 hours post feeding, the rest of the ticks were allowed to feed to repletion. The mice were then euthanized at 7 or 14 days after tick feeding, and the blood, the feeding site of the skin, the tibiotarsus joints, bladder, ears, and heart were collected.

### Quantification of *cspA* expression levels using quantitative RT-PCR

The *B*. *burgdorferi* strain B31-5A15, ticks, or mouse tissues were mixed with glass beads and then homogenized by a Precellys 24 High-Powered Bead Mill Homogenizer (Bertin, Rockville, MD). RNA was extracted from these homogenized bacteria, ticks or tissue samples using Direct-Zol RNA MiniPrep Plus Kit (Zymo Research, Irvine, CA) according to the manufacturer’s instructions. Contaminating DNA was removed using RQ1 RNase-Free DNase (Promega, Madison, WI) following vendor’s instruction. cDNA was synthesized from 1 μg of RNA measured by spectrophotometer using a qScript cDNA SuperMix (Quanta Bioscience, Beverly, MA) according to the manufacturer’s instructions. Then, the quantification of *cspA*, *flaB*, or *rec*A expression from cDNA was performed using an Applied Biosystems 7500 Real-Time PCR system (ThermoFisher) in conjunction with PowerUp SYBR Green Master Mix (ThermoFisher), based on amplification of the *B*. *burgdorferi cspA*, *flaB*, or *recA* gene using primers BBCspAfp and BBCspArp (for *cspA*), BBFlaBfp and BBFlaBrp (for *flaB*), or BBRecAfp and BBRecArp (for *recA*) as described previously [94] (S3 Table), respectively. Cycling parameters for SYBR green-based reactions were 50°C for 2 minutes, 95°C for 10 minutes, 45 cycles of 95°C for 15 seconds, and 60°C for 1 minute. Melting curve analysis for purity was performed on each sample by performing 80 cycles of increasing temperature for 10 seconds, each beginning at 55°C. Three biological replicates were included. Each biological replicate was run in duplicates and checked for intra-run variation. The gene expression of *cspA* or *recA* was normalized to that of *flaB* using the ΔCT method, where the relative expression of target (*cspA* or *recA*), normalized to the expression of *flaB*, is given by 2^−ΔCT^, where Ct is the cycle number of the detection threshold (see Eq 2). All analyses and calculations were performed using the Applied Biosystem sequence detection software version 7.5.1 (ThermoFisher).

$$Relative\;cspA\;or\;recA\;expression\;to\;flab\;=\;2^{-(Ct\left(flaB\right)-Ct(cspA\;or\;recA))}$$ (2)

### Determination of CspA production levels of *B*. *burgdorferi* in ticks using flow cytometry

Ticks mixed with Enzyme Free cell dissociation buffer (ThermoFisher) were gently disrupted by pipette tips and then incubated at 37°C for 30 minutes to release the spirochetes into the buffer. The ticks-spirochetes mixtures were subsequently spun down, washed by PBS, and permeabilized by incubating the mixture with 100% methanol for one hour. After these mixtures were washed three times with HBSC buffer containing glucose and BSA, they were incubated with mouse antisera raised against CspA_B31_ (1:250x) or monoclonal mouse antibody against FlaB (1:250x) as primary antibody followed by an Alexa 647-conjugated goat anti-mouse IgG (1:250x) as a secondary antibody. Three hundred microliters of formalin (0.1%) were then added for fixing. The spirochetes were first sorted using a FACSAria cell sorter II equipped with FACSDiva software (BD Bioscience). The purity of sorted populations was greater than 70% in all experiments (S8 Fig). Then, the production of CspA and FlaB (negative control) in these spirochetes was measured by this equipment and software. The mean fluorescence index (MFI) of each sample was obtained from FlowJo software representing the production of the indicated proteins. The “ΔMFI” values are the mean fluorescence index obtained from each of these strains subtracting that obtained from the strains stained only by the secondary antibody. The production levels of CspA and FlaB in different stages of the enzootic cycle and *in vitro* cultured *B*. *burgdorferi* were presented as “ΔMFI” (Fig 5C). These results represent the mean of three independent determinations ± the standard deviation of mean.

### Quantification of *B*. *burgdorferi* in infected ticks, tissues and blood samples

The ticks collected from the chambers on the mice were mixed with glass beads and then homogenized by a Precellys 24 High‑Powered Bead Mill Homogenizer (Bertin, Rockville, MD). The DNA from mouse tissues or blood or homogenized ticks was extracted using the EZ-10 Genomic DNA kit (for mouse tissues and blood, Biobasic, Amherst, New York) or the insect DNA kit (for ticks, OMEGA Biotek, Norcross, GA). The quantity and quality of DNA for each tissue sample have been assessed by measuring the concentration of DNA and the ratio of the UV absorption at 280 to 260 using a nanodrop 1000 UV/Vis spectrophotometer (ThermoFisher). The amount of DNA used in this study was 100 ng for each sample, and the 280:260 ratio was between 1.75 to 1.85, indicating the lack of contaminating RNA or proteins. Quantitative PCR (qPCR) was then performed to quantitate bacterial loads, using 100 ng of DNA per reaction. *B*. *burgdorferi* genomic equivalents were calculated using an Applied Biosystems 7500 Real-Time PCR system (ThermoFisher) in conjunction with PowerUp SYBR Green Master Mix (ThermoFisher), based on amplification of the *B*. *burgdorferi recA* gene using primers BBRecAfp and BBRecArp (S3 Table), as described previously [77]. Cycling parameters for SYBR green-based reactions were 50°C for 2 minutes, 95°C for 10 minutes, 45 cycles of 95°C for 15 seconds, and 60°C for 1 minute. The number of *recA* copies was calculated by establishing a threshold cycle (Ct) standard curve of a known number of *recA* gene extracted from *B*. *burgdorferi* strain B31, then comparing the Ct values of the experimental samples. To determine that the shuttle vectors expressing CspA variants is not missing during tick-mouse studies, the *colE1* gene on the shuttle vector was amplified using primers BBColE1fp and BBColE1rp (S3 Table) and the same cycling parameters used to amplify *recA* described above. The bacterial burdens determined using *colE1* primers were compared with the burdens obtained using primers to amplify the *recA* gene, the gene on the chromosome of spirochetes. The shuttle vectors were not missing as the bacterial loads determined using *colE1* primers is close to 100% of the bacterial loads determined using *recA* primers. To assure the low signals were not simply a function of the presence of PCR inhibitors in the DNA preparation, we subjected 5 samples from blood, tibiotarsus joints, and bladder of the mice infected by *B*. *burgdorferi* strain B31-5A15, B31-5A4NP1Δ*cspA*-V or this *cspA* mutant strain producing CspA_B31_, CspA_PKo_, CspA_ZQ1_, or CspA_B31_L246D to qPCR using mouse nidogen primers mNidfp and mNidrp (S3 Table) as an internal standard [77]. As predicted, we detected 10^7^ copies of the nidogen gene from 100ng of each DNA sample, ruling out the presence of PCR inhibitors in these samples.

### Statistical analysis

Significant differences between samples were determined using Student’s T test or the one-way ANOVA with post hoc Bonferroni correction. P-values were determined for each sample. A P-value < 0.05 (*) or (^#^) was considered to be significant.

## Supporting information

S1 Fig Sequence alignment of CspA variants from *B*. *burgdorferi* sensu lato. The amino acid sequences of CspA variants from *B*. *burgdorferi* strains B31 (*Bburg*_B31_BBA68) and ZS7 (*Bburg_*ZS7*_*A59), *B*. *afzelii* strains MMS (*Bafz_*MMS_MMSA71) and PKo (*Bafz_*PKo_A0067), *B*. *garinii* strain ZQ1 (*Bgar_*ZQ1_ZQA68), *B*. *bavariensis* strain PBi (*Bbav_*PBi_BGA66), and *B*. *spielmanii* strain A14S (*Bspiel_*A14S_A0067) were aligned using M-Coffee with default parameters. Black shaded residues are identical among all of these variants. The red asterisk indicates the leucine-246 of CspA_B31_, which is required for human, mouse, horse, and quail FH-binding activity of this protein.
(TIF)

S2 Fig Recombinant CspA variants or mutants display variable binding ability to FH from different vertebrate animals by ELISA. The indicated concentrations of various recombinant histidine-tagged CspA_B31_ (“B31”), CspA_PKo_ (“PKo”), CspA_ZQ1_ (“ZQ1”), CspA_B31_L246D (“L246D”), or DbpA (“DbpA”, negative control) were added to triplicate wells coated with FH from **(A)** human, **(B)** mouse, **(C)** horse, or **(D)** quail, and protein binding was quantitated by ELISA. The experiments were performed on three independent occasions; within each occasion, samples were run in duplicate. All experiments were performed with a single preparation of recombinant proteins. Shown is a representative experiment from the average of two replicates. The K_D_ values (Table 1) representing the FH-binding affinity of each protein were determined from the average of three experiments.
(TIF)

S3 Fig Recombinant CspA variants or mutants exhibit variable binding ability to FH from different vertebrate animals by SPR. Ten micrograms of FH from **(A to C)** human, **(D to F)** mouse, or **(G and H)** horse were conjugated on a SPR chip, resulting the response unit (RU) as 563, 511, and 433 for human, mouse, and horse FH, respectively. Different concentrations (0.125 to 2 or 4μM) of histidine tagged CspA_B31_ (“B31”), CspA_PKo_ (“PKo”), CspA_ZQ1_ (“ZQ1”), or CspA_B31_L246D (“L246D”) were flowed over a surface of the chip. Binding was measured in response units (RU) by SPR (see Materials and methods). The experiments were performed on three independent occasions; within each occasion, samples were run in duplicate. All experiments were performed with a single preparation of recombinant proteins. The k_on_, k_off_, and K_D_ values (Table 1) were determined from the average of these three experiments. Panel A, D, and F are representative experiments applying 1 μM of indicated CspA proteins to the chip coated with FH from indicated species.
(TIF)

S4 Fig The mutation of leucine-246 to aspartate of CspA_B31_ does not affect its structure. Far-UV CD analysis of CspA_B31_ and CspA_B31_L246D. The molar ellipticity, Φ, was measured from 190 to 250 nm for 10μM of each protein in PBS. The predicted spectrum of CspA_B31_ was generated applying the full-length amino acid sequences of this protein to DichroWeb (http://dichroweb.cryst.bbk.ac.uk/html/links.shtml).
(TIF)

S5 Fig CspA variants differ in their ability to confer spirochete binding to FH from C3-depleted human or mouse serum. *B*. *burgdorferi* strain B31-5A15 (“B31-5A15”), B31-5A4NP1Δ*cspA* harboring the vector pBSV2G (“Δ*cspA*/Vector”), or this *cspA* mutant strain producing CspA_B31_ (“Δ*cspA*/pCspA_B31_”), CspA_PKo_ (“Δ*cspA*/pCspA_PKo_”), CspA_ZQ1_ (“Δ*cspA*/pCspA_ZQ1_”), or CspA_B31_L246D (“Δ*cspA*/pCspA_B31_L246D”), or B313 carrying the vector pBSV2G (“B313/Vector”, negative control) was incubated with C3-depleted human or mouse serum. The bacteria were stained with a sheep anti-FH polyclonal IgG followed by an Alexa 647-conjugated donkey anti-sheep IgG prior to being applied to flow cytometry analysis. **(Left panel)** Representative histograms of flow cytometry analysis showing the levels of FH from **(A)** human or **(B)** mouse binding to indicated *B*. *burgdorferi* strains. **(Right panel)** The levels of *B*. *burgdorferi* binding to FH from **(A)** human or **(B)** mouse were measured by flow cytometry and presented as mean fluorescence index (MFI). Each bar represents the mean of three independent determinations ± SEM. Significant differences (P < 0.05 by one-way ANOVA with post hoc Bonferroni correction) in the levels of FH binding relative to the B313/Vector (“*”) or between two strains relative to each other (“#”).
(TIF)

S6 Fig The mutation of leucine-246 to aspartate of CspA_B31_ does not affect its ability in binding to C7, C9, or plasminogen. Ten micrograms of human **(A and B)** C7, **(C and D)** C9, or **(E and F)** plasminogen (PLG) were conjugated on a SPR chip, resulting the response unit (RU) as 2823, 2034, and 2106 for C7, C9, and PLG, respectively. Different concentrations (0.0625 to 1 μM) of histidine tagged **(A, C, and E)** CspAB31 (“B31”) or **(B, D and F)** CspA_B31_L246D (“L246D”) were flowed over the chip. Binding was measured in RU by SPR (see Materials and methods). The experiments were performed on three independent occasions; within each occasion, samples were run in duplicate. All experiments were performed with a single preparation of recombinant proteins. The k_on_, k_off_, and K_D_ values (S1 Table) were determined from the average of these three experiments.
(TIF)

S7 Fig Summary of experimental design using a mouse model to test the role of CspA in the enzootic cycle. Experimental infection of **(A)** C3H/HeN mice using needle infection **(B)** C3H/HeN mice or **(C)** BALB/c or BALB/c C3^-/-^ mice by larvae acquisition and nymph transmission.
(TIF)

S8 Fig FACS sorting of *B*. *burgdorferi* from ticks. *I*. *scapularis* ticks carrying *B*. *burgdorferi* strain B31-5A15 were disrupted by pipette tips followed by incubation with enzyme free cell dissociation buffer. The resulting suspensions were subjected to FACS to isolate *B*. *burgdorferi*. To enhance the sorting efficiency, the spirochetes were permeabilized by methanol and then stained by a mouse monoclonal antibody against *B*. *burgdorferi* flagellin (FlaB) followed by Alexa 647 conjugated antibody against mouse IgG. Shown is the FACS data from gating for *B*. *burgdorferi* in the suspensions of disrupted nymphal ticks at 24 hours post fed on naïve C3H/HeN mice. **(A)** To discriminate between *B*. *burgdorferi* and debris of ticks or aggregations, the *B*. *burgdorferi*-tick suspensions were introduced into FACS and plotted by forward scattering (FSC) or side scattering (SSC). Two populations with distinct FSC and SSC (R1 and R2) were sorted. **(B)** The populations of R1 and R2 from panel A were examined for their fluorescence intensity of Alexa 647. The majority of the cells in R1 was Alexa 647 positive whereas the population in R2 was Alexa 647 negative, suggesting *B*. *burgdorferi* was sorted at R1. **(C)** The same disrupted ticks-*B*. *burgdorferi* suspensions were also permeabilized and then stained by Alexa 647 conjugated antibody against mouse IgG but not antibody against *B*. *burgdorferi* flagellin (FlaB) to detect the background staining. The same populations of R1 and R2 were also gated. **(D)** Both R1 and R2 from panel C were Alexa 647 negative, indicating low levels of background staining for each of these two populations. The **(E)** pre- and **(F)** post-sorted cells were also imaged under a dark-field microscope (40x, bar = 5μm). The arrows indicate *B*. *burgdorferi* spirochetes.
(TIF)

S9 Fig The isogenic *B*. *burgdorferi* strain producing no CspA or each of the CspA variants colonizes distal mammalian tissues at similar levels as WT strain during needle infection. C3H/HeN mice were infected by needles with 10^5^ *B*. *burgdorferi* strains B31-5A15 (“B31-5A15”), B31-5A4NP1Δ*cspA* harboring the vector pBSV2G (“Δ*cspA*/Vector”), or this *cspA* mutant strain producing CspA_B31_ (“Δ*cspA*/pCspA_B31_”), CspA_PKo_ (“Δ*cspA*/pCspA_PKo_”), CspA_ZQ1_ (“Δ*cspA*/pCspA_ZQ1_”), or CspA_B31_L246D (“Δ*cspA*/pCspA_B31_L246D”). At 14 days post infection, the bacterial loads in **(A)** the inoculation site of skin (“inoc. Site”), **(B)** tibiotarsus joints, **(C)** heart, **(D)** bladder, and **(E)** ears were determined by qPCR and normalized to 100 ng total DNA. Shown are the geometric mean of bacterial loads ± 95% confidence interval of 5 mice per group. No Significant differences (P > 0.05 by one-way ANOVA with post hoc Bonferroni correction) of bacterial burdens was observed in these tissues from the mice infected by each of those *B*. *burgdorferi* strains.
(TIF)

S10 Fig Needle inoculation of a *cspA* deficient or WT *B*. *burgdorferi* results in similar levels of tissue colonization and bacteremia at early stages of infection. C3H/HeN mice were infected via needles with 10^5^ *B*. *burgdorferi* strains B31-5A15 (“5A15”) or B31-5A4NP1Δ*cspA* harboring the vector pBSV2G (“5A4NP1Δ*cspA*/Vector”). At 4 days post infection (“4dpi”), the bacterial loads in the inoculation site of skin (“Inoc. Site”) and blood were determined by qPCR and normalized to 100 ng total DNA. Shown are the geometric mean of bacterial loads ± 95% confidence interval of 5 mice per group. No Significant differences (P > 0.05 by Student’s t test) of bacterial burdens were observed from the mice infected between these *B*. *burgdorferi* strains.
(TIF)

S11 Fig A *cspA*-deficient *B*. *burgdorferi* is cleared in fed nymphs at 48 and 72 hours post feeding. The nymphs infected with *B*. *burgdorferi* strain B31-5A15 (“5A15”) or a *cspA* mutant strain B31-5A4NP1Δ*cspA* harboring the vector pBSV2G (“5A4NP1Δ*cspA*/Vector”) were allowed to feed on C3H/HeN mice for 48 and 72 hours (“48hpf” and “72hpf”). Bacterial loads in those fed nymphs were determined by qPCR. Shown are the geometric mean of bacterial loads ± 95% confidence interval of four nymphs (for nymphs carrying strain B31-5A15) or five nymphs (for nymphs carrying the strain B31-5A4NP1Δ*cspA* harboring the vector) per group. “*” indicates statistically (P < 0.05 by Student’s t test) different between two different strains-infected nymphs.
(TIF)

S12 Fig A *cspA* deficient *B*. *burgdorferi* is incapable of colonizing distal mammalian tissues after the infection by nymphal ticks. C3H/HeN mice were infected by needles with 10^5^ *B*. *burgdorferi* strain B31-5A15 (“5A15”) or B31-5A4NP1Δ*cspA* harboring the vector pBSV2G (“5A4NP1Δ*cspA*/Vector”). At 14 days post infection, the uninfected *I*. *scapularis* larval ticks were allowed to feed on each of these mice to repletion. After the replete larvae molt into nymphs, those *B*. *burgdorferi*-infected nymphs were allowed to feed on naïve C3H/HeN mice to repletion. At 14 days after nymphal tick feeding (“14dpf”), the bacterial loads in the inoculation site of skin (“inno. site”), tibiotarsus and knee joints, bladder, and heart were determined by qPCR and normalized to 100 ng total DNA. Shown are the geometric mean of bacterial loads ± 95% confidence interval of four mice per group. Significant differences (P < 0.05, Student’s t test) in the spirochete burdens between two strains relative to each other (“*”).
(TIF)

S13 Fig The antisera of CspA_B31_, CspA_PKo_, CspA_ZQ1_, CspA_B31_L246D recognize each of these CspA variants or mutant proteins at indistinguishable levels. One microgram of CspA_B31_, CspA_PKo_, CspA_ZQ1_, or CspA_B31_L246D, or BSA (as a negative control) was coated on triplicate wells in a microtiter plate. The anti-sera raised against **(A)** CspA_B31_ (“αCspA_B31_”), **(B)** CspA_PKo_ (“αCspA_PKo_”), **(C)** CspA_ZQ1_ (“αCspA_ZQ1_”) or **(D)** pre-immune serum (“Preimmune”, negative control) were added to these wells. Bound antibody was measured by ELISA (see Materials and methods), and the mean OD_405_ values ± standard deviations were determined. Asterisk (“*”) indicates significant different levels of recognition by particular anti-sera to CspA proteins (P < 0.05) compared that to BSA determined by one-way ANOVA with post hoc Bonferroni correction.
(TIF)

S1 Table CspAB31 and CspAB31L246D display indistinguishable affinity in binding to C7, C9 and plasminogen. (PDF)

S2 Table *B*. *burgdorferi* strains and DNA plasmids used in this study. (PDF)

S3 Table Primers used in this study. (PDF)
