# Supplementary figures and images for: Polymorphic factor H-binding activity of CspA protects Lyme borreliae from the host complement in feeding ticks to facilitate tick-to-host transmission
Source: PLoS Pathog. 2018 May 29;14(5):e1007106. doi: 10.1371/journal.ppat.1007106 (PMC5993331; doi:10.1371/journal.ppat.1007106)

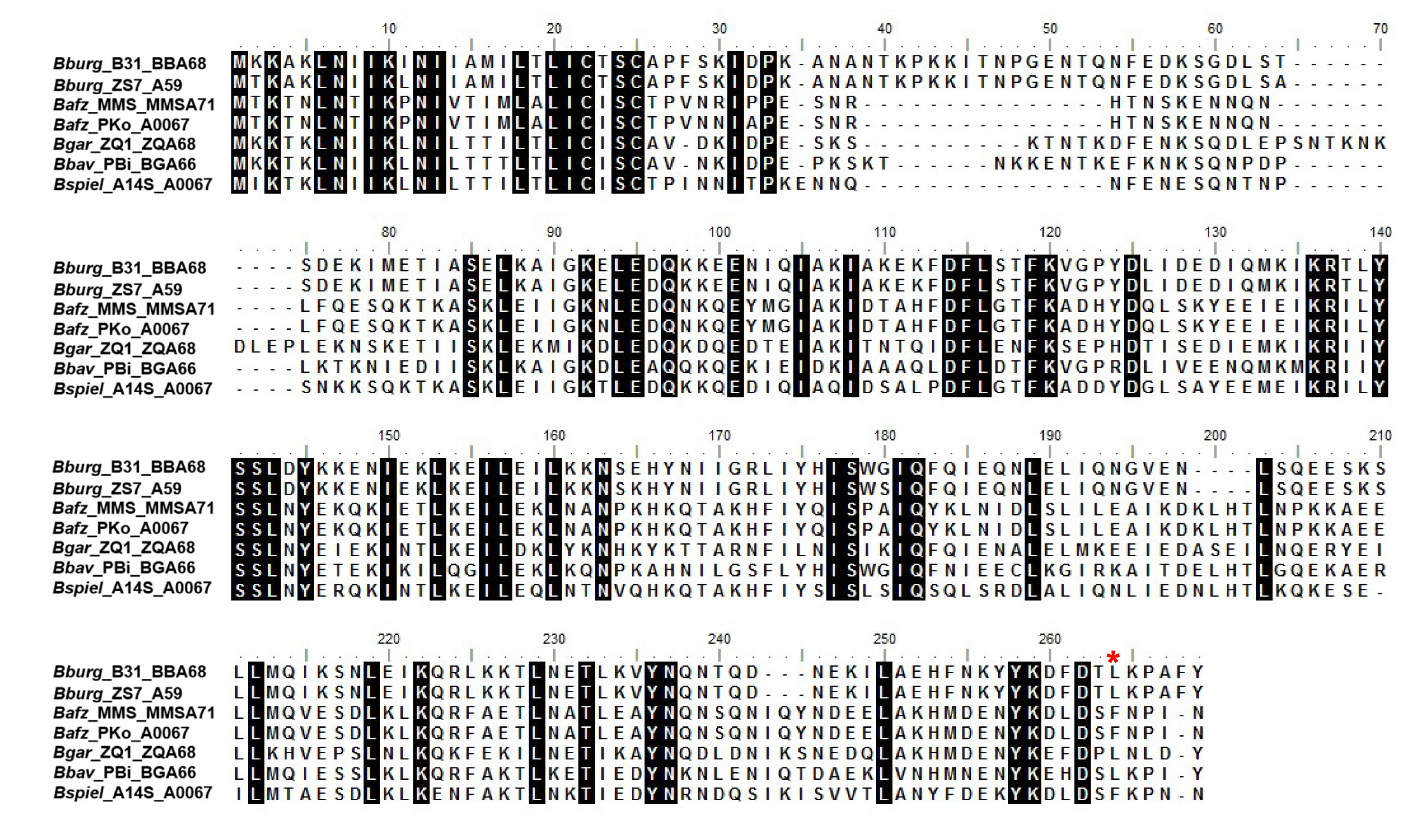

Supplement: S1 Fig — The amino acid sequences of CspA variants from B. burgdorferi strains B31 (Bburg_B31_BBA68) and ZS7 (Bburg_ZS7_A59), B. afzelii strains MMS (Bafz_MMS_MMSA71) and PKo (Bafz_PKo_A0067), B. garinii strain ZQ1 (Bgar_ZQ1_ZQA68), B. bavariensis strain PBi (Bbav_PBi_BGA66), and B. spielmanii strain A14S (Bspiel_A14S_A0067) were aligned using M-Coffee with default parameters. Black shaded residues are identical among all of these variants. The red asterisk indicates the leucine-246 of CspAB31, which is required for human, mouse, horse, and quail FH-binding activity of this protein. (TIF) [file ppat.1007106.s001.tif]

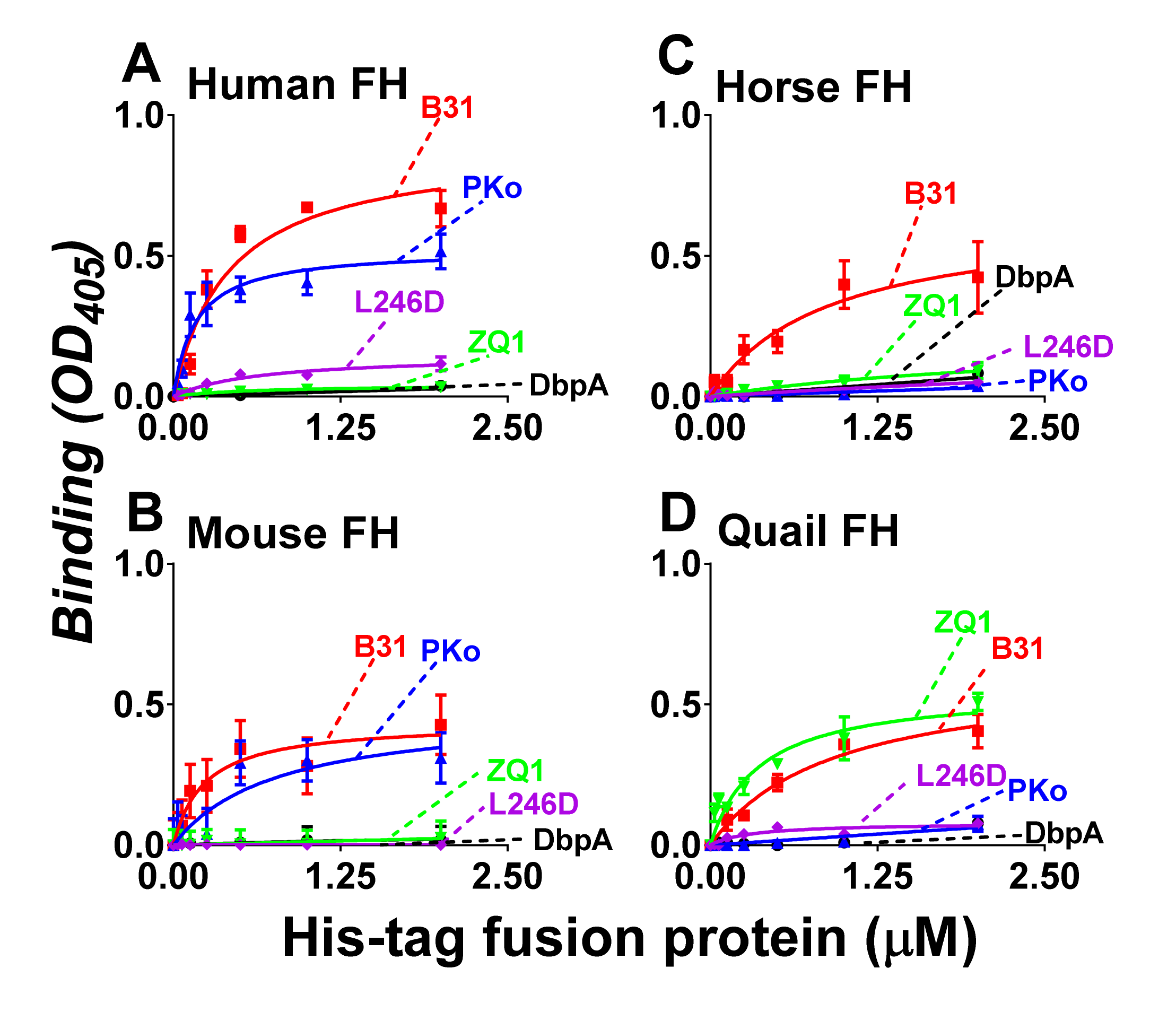

Supplement: S2 Fig — The indicated concentrations of various recombinant histidine-tagged CspAB31 (“B31”), CspAPKo (“PKo”), CspAZQ1 (“ZQ1”), CspAB31L246D (“L246D”), or DbpA (“DbpA”, negative control) were added to triplicate wells coated with FH from (A) human, (B) mouse, (C) horse, or (D) quail, and protein binding was quantitated by ELISA. The experiments were performed on three independent occasions; within each occasion, samples were run in duplicate. All experiments were performed with a single preparation of recombinant proteins. Shown is a representative experiment from the average of two replicates. The KD values (Table 1) representing the FH-binding affinity of each protein were determined from the average of three experiments. (TIF) [file ppat.1007106.s002.tif]

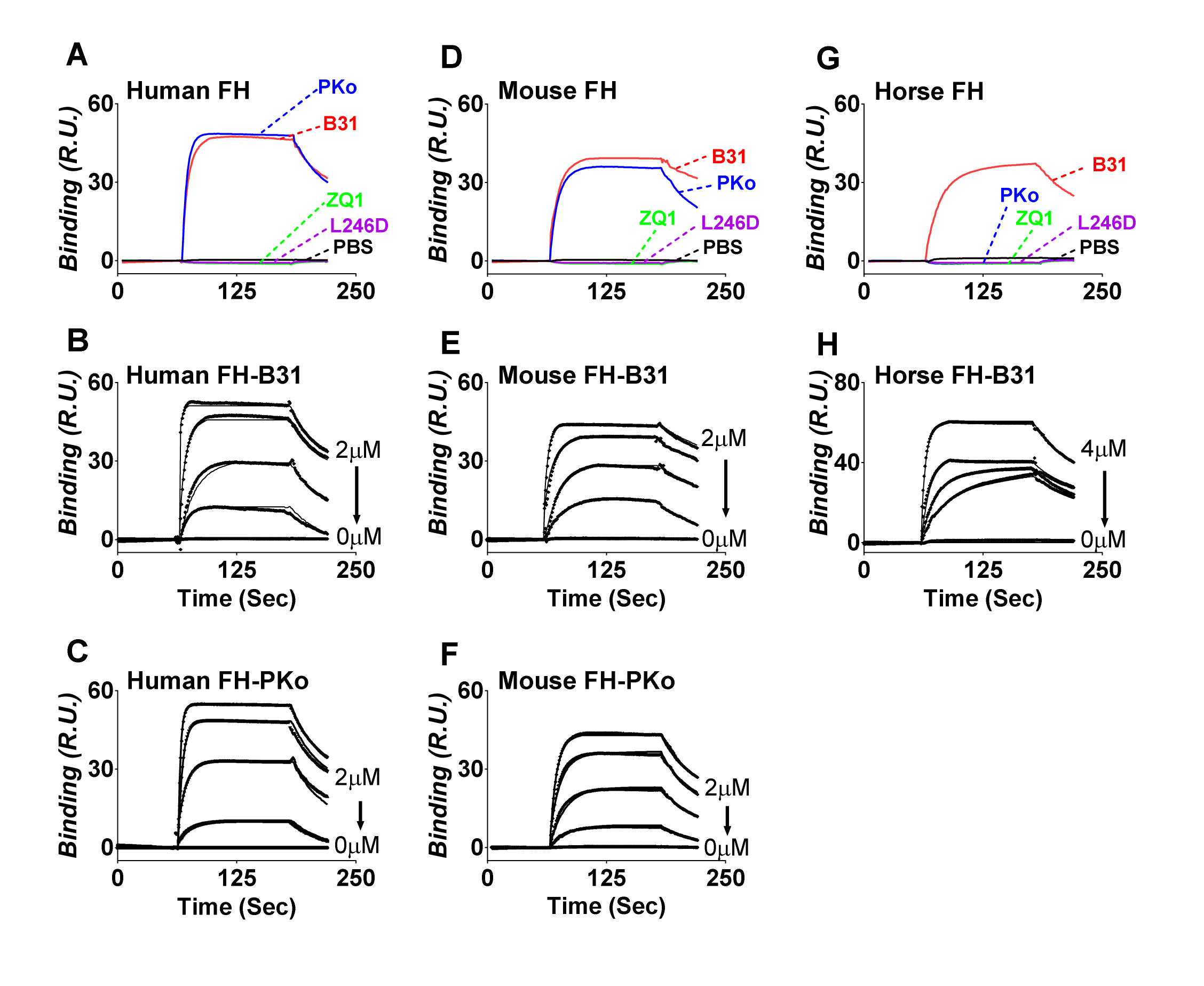

Supplement: S3 Fig — Ten micrograms of FH from (A to C) human, (D to F) mouse, or (G and H) horse were conjugated on a SPR chip, resulting the response unit (RU) as 563, 511, and 433 for human, mouse, and horse FH, respectively. Different concentrations (0.125 to 2 or 4μM) of histidine tagged CspAB31 (“B31”), CspAPKo (“PKo”), CspAZQ1 (“ZQ1”), or CspAB31L246D (“L246D”) were flowed over a surface of the chip. Binding was measured in response units (RU) by SPR (see Materials and methods). The experiments were performed on three independent occasions; within each occasion, samples were run in duplicate. All experiments were performed with a single preparation of recombinant proteins. The kon, koff, and KD values (Table 1) were determined from the average of these three experiments. Panel A, D, and F are representative experiments applying 1 μM of indicated CspA proteins to the chip coated with FH from indicated species. (TIF) [file ppat.1007106.s003.tif]

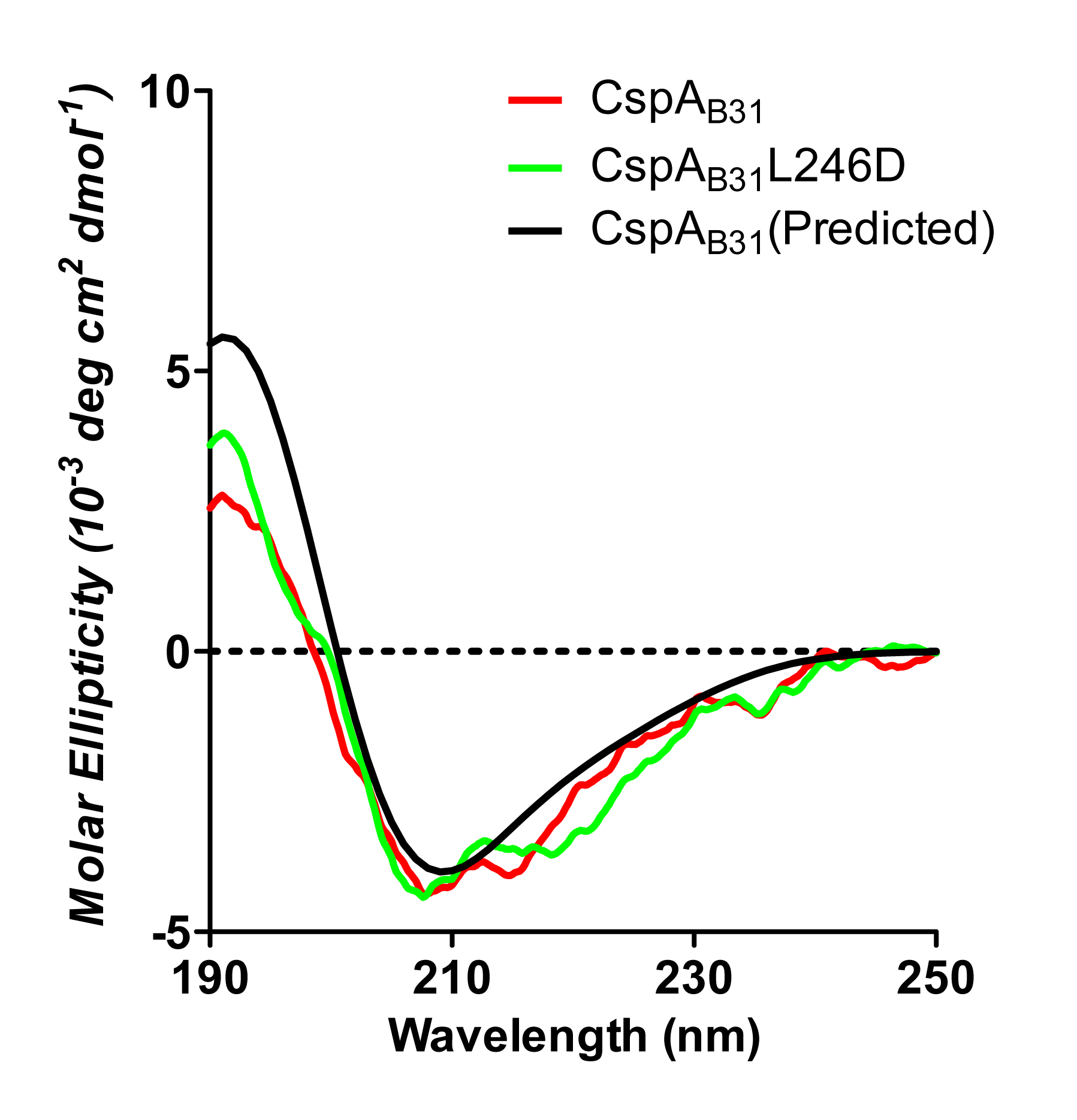

Supplement: S4 Fig — Far-UV CD analysis of CspAB31 and CspAB31L246D. The molar ellipticity, Φ, was measured from 190 to 250 nm for 10μM of each protein in PBS. The predicted spectrum of CspAB31 was generated applying the full-length amino acid sequences of this protein to DichroWeb (http://dichroweb.cryst.bbk.ac.uk/html/links.shtml). (TIF) [file ppat.1007106.s004.tif]

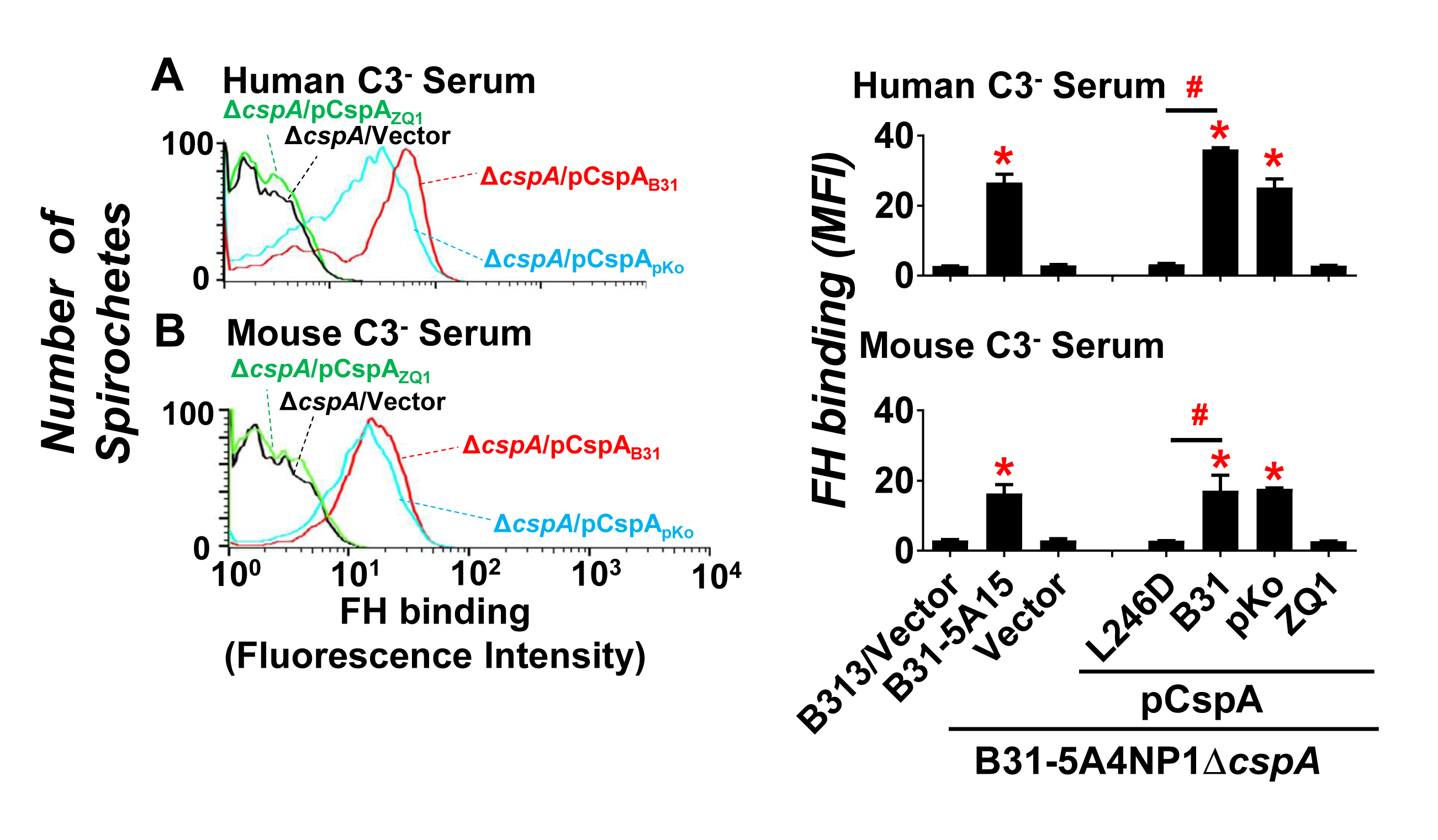

Supplement: S5 Fig — B. burgdorferi strain B31-5A15 (“B31-5A15”), B31-5A4NP1ΔcspA harboring the vector pBSV2G (“ΔcspA/Vector”), or this cspA mutant strain producing CspAB31 (“ΔcspA/pCspAB31”), CspAPKo (“ΔcspA/pCspAPKo”), CspAZQ1 (“ΔcspA/pCspAZQ1”), or CspAB31L246D (“ΔcspA/pCspAB31L246D”), or B313 carrying the vector pBSV2G (“B313/Vector”, negative control) was incubated with C3-depleted human or mouse serum. The bacteria were stained with a sheep anti-FH polyclonal IgG followed by an Alexa 647-conjugated donkey anti-sheep IgG prior to being applied to flow cytometry analysis. (Left panel) Representative histograms of flow cytometry analysis showing the levels of FH from (A) human or (B) mouse binding to indicated B. burgdorferi strains. (Right panel) The levels of B. burgdorferi binding to FH from (A) human or (B) mouse were measured by flow cytometry and presented as mean fluorescence index (MFI). Each bar represents the mean of three independent determinations ± SEM. Significant differences (P < 0.05 by one-way ANOVA with post hoc Bonferroni correction) in the levels of FH binding relative to the B313/Vector (“*”) or between two strains relative to each other (“#”). (TIF) [file ppat.1007106.s005.tif]

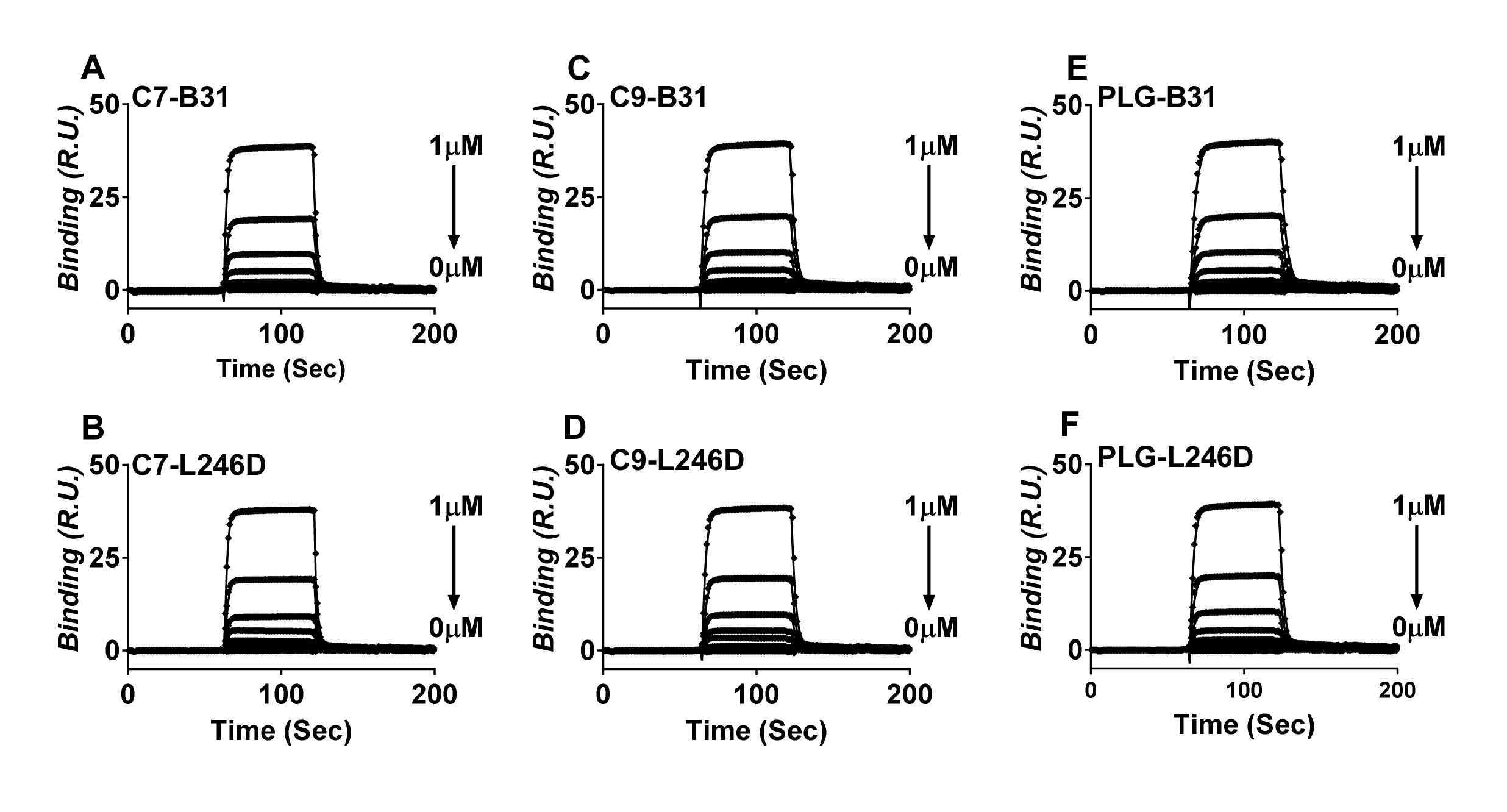

Supplement: S6 Fig — Ten micrograms of human (A and B) C7, (C and D) C9, or (E and F) plasminogen (PLG) were conjugated on a SPR chip, resulting the response unit (RU) as 2823, 2034, and 2106 for C7, C9, and PLG, respectively. Different concentrations (0.0625 to 1 μM) of histidine tagged (A, C, and E) CspAB31 (“B31”) or (B, D and F) CspAB31L246D (“L246D”) were flowed over the chip. Binding was measured in RU by SPR (see Materials and methods). The experiments were performed on three independent occasions; within each occasion, samples were run in duplicate. All experiments were performed with a single preparation of recombinant proteins. The kon, koff, and KD values (S1 Table) were determined from the average of these three experiments. (TIF) [file ppat.1007106.s006.tif]

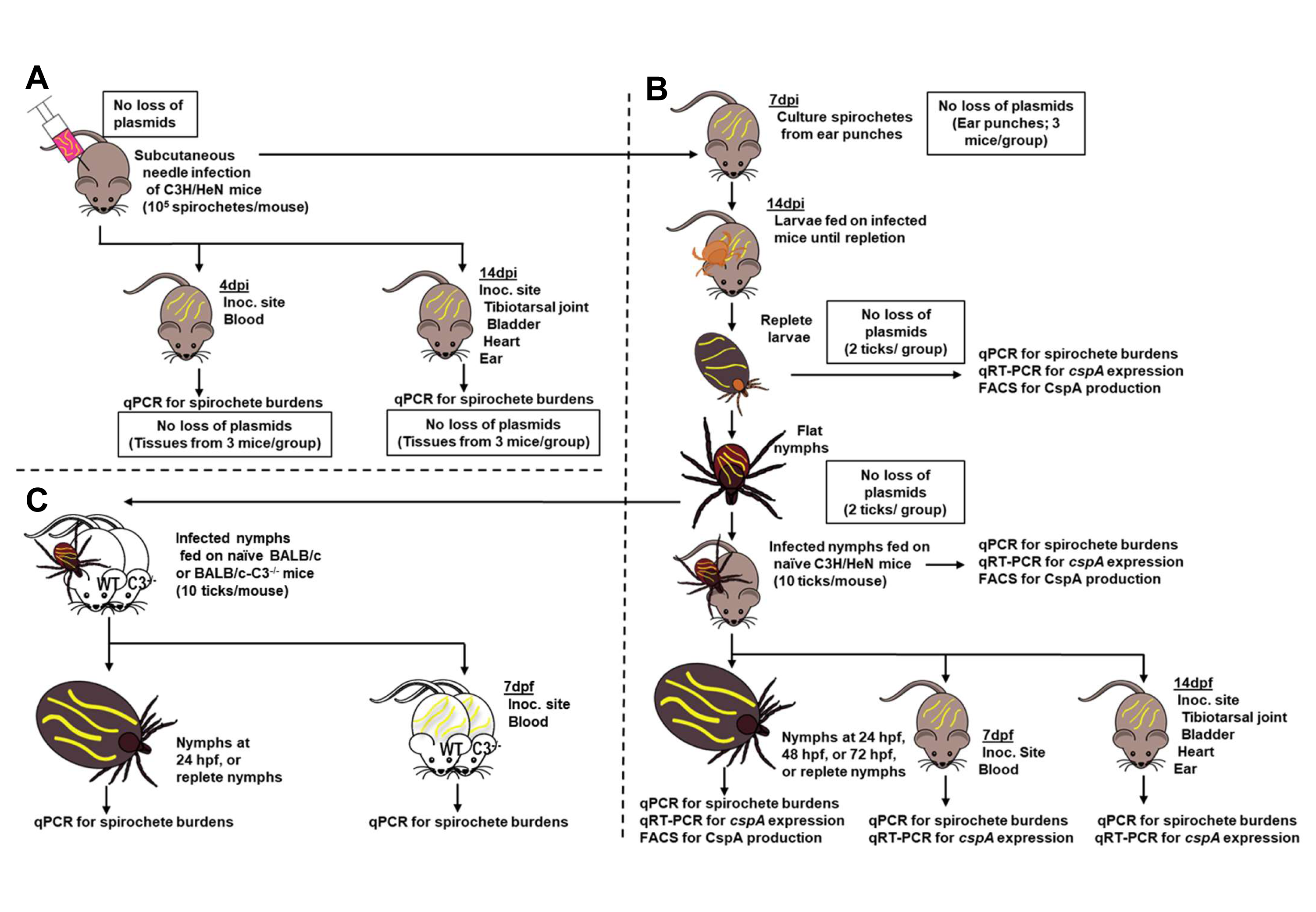

Supplement: S7 Fig — Experimental infection of (A) C3H/HeN mice using needle infection (B) C3H/HeN mice or (C) BALB/c or BALB/c C3-/- mice by larvae acquisition and nymph transmission. (TIF) [file ppat.1007106.s007.tif]

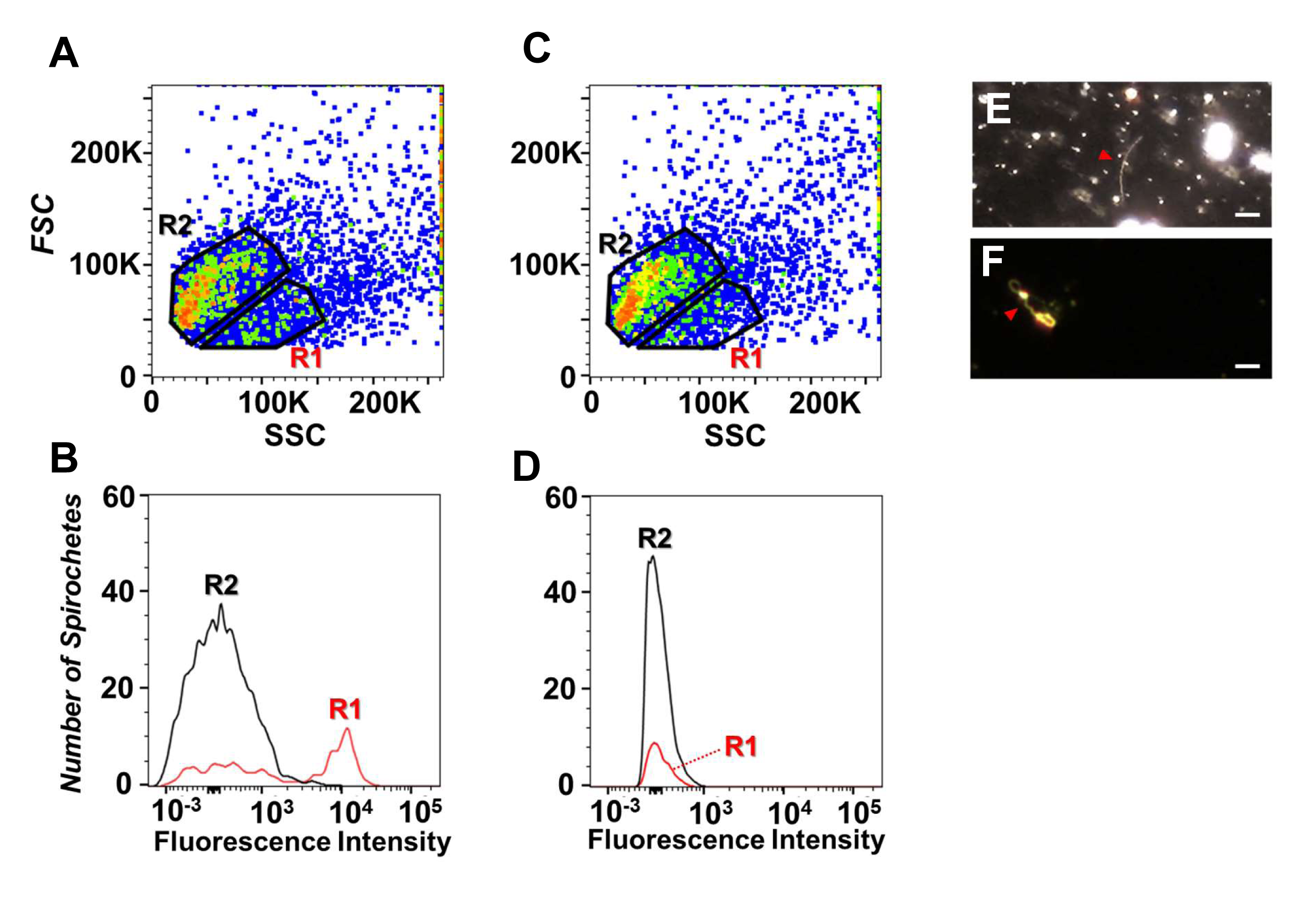

Supplement: S8 Fig — I. scapularis ticks carrying B. burgdorferi strain B31-5A15 were disrupted by pipette tips followed by incubation with enzyme free cell dissociation buffer. The resulting suspensions were subjected to FACS to isolate B. burgdorferi. To enhance the sorting efficiency, the spirochetes were permeabilized by methanol and then stained by a mouse monoclonal antibody against B. burgdorferi flagellin (FlaB) followed by Alexa 647 conjugated antibody against mouse IgG. Shown is the FACS data from gating for B. burgdorferi in the suspensions of disrupted nymphal ticks at 24 hours post fed on naïve C3H/HeN mice. (A) To discriminate between B. burgdorferi and debris of ticks or aggregations, the B. burgdorferi-tick suspensions were introduced into FACS and plotted by forward scattering (FSC) or side scattering (SSC). Two populations with distinct FSC and SSC (R1 and R2) were sorted. (B) The populations of R1 and R2 from panel A were examined for their fluorescence intensity of Alexa 647. The majority of the cells in R1 was Alexa 647 positive whereas the population in R2 was Alexa 647 negative, suggesting B. burgdorferi was sorted at R1. (C) The same disrupted ticks-B. burgdorferi suspensions were also permeabilized and then stained by Alexa 647 conjugated antibody against mouse IgG but not antibody against B. burgdorferi flagellin (FlaB) to detect the background staining. The same populations of R1 and R2 were also gated. (D) Both R1 and R2 from panel C were Alexa 647 negative, indicating low levels of background staining for each of these two populations. The (E) pre- and (F) post-sorted cells were also imaged under a dark-field microscope (40x, bar = 5μm). The arrows indicate B. burgdorferi spirochetes. (TIF) [file ppat.1007106.s008.tif]

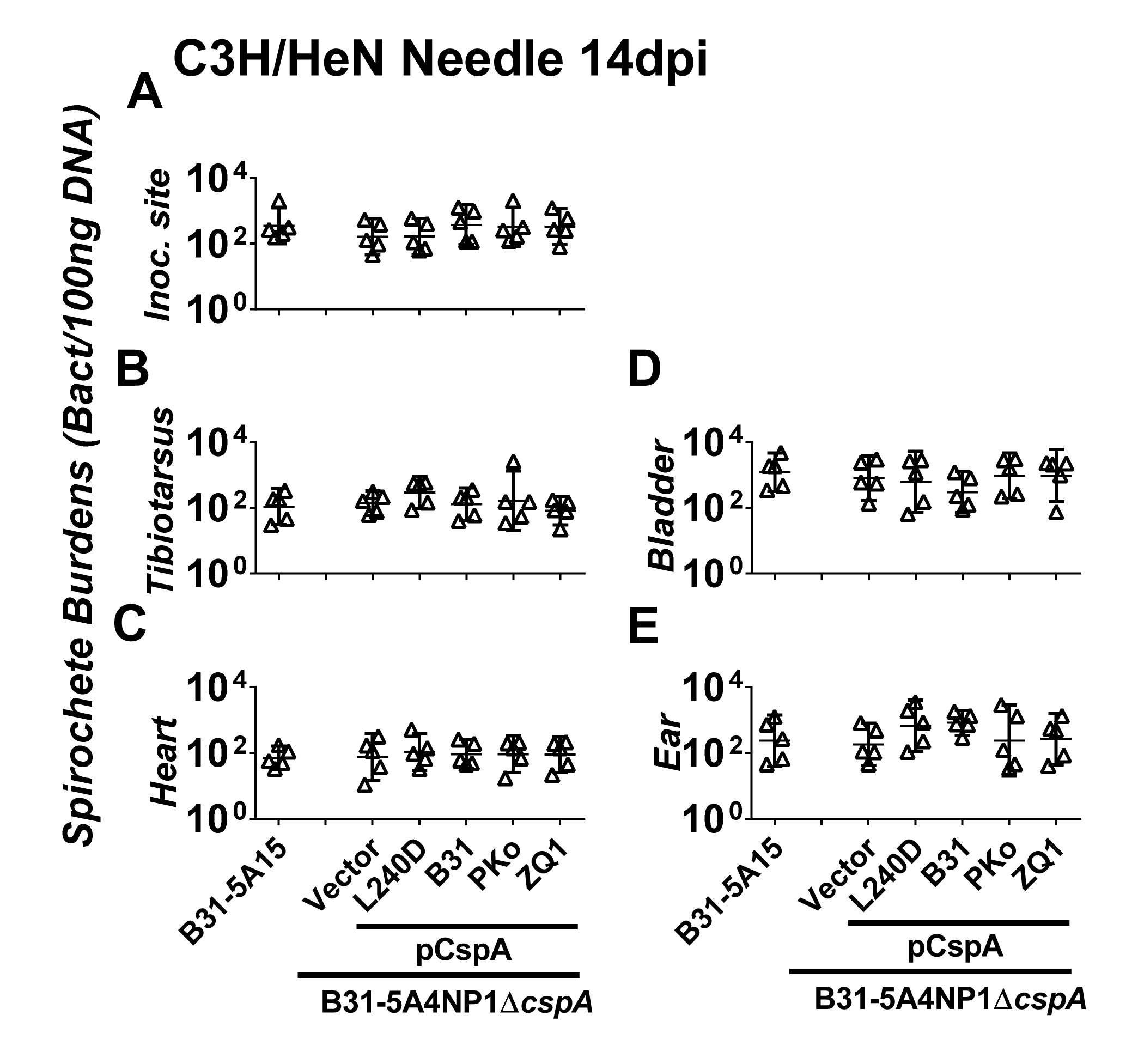

Supplement: S9 Fig — C3H/HeN mice were infected by needles with 105 B. burgdorferi strains B31-5A15 (“B31-5A15”), B31-5A4NP1ΔcspA harboring the vector pBSV2G (“ΔcspA/Vector”), or this cspA mutant strain producing CspAB31 (“ΔcspA/pCspAB31”), CspAPKo (“ΔcspA/pCspAPKo”), CspAZQ1 (“ΔcspA/pCspAZQ1”), or CspAB31L246D (“ΔcspA/pCspAB31L246D”). At 14 days post infection, the bacterial loads in (A) the inoculation site of skin (“inoc. Site”), (B) tibiotarsus joints, (C) heart, (D) bladder, and (E) ears were determined by qPCR and normalized to 100 ng total DNA. Shown are the geometric mean of bacterial loads ± 95% confidence interval of 5 mice per group. No Significant differences (P > 0.05 by one-way ANOVA with post hoc Bonferroni correction) of bacterial burdens was observed in these tissues from the mice infected by each of those B. burgdorferi strains. (TIF) [file ppat.1007106.s009.tif]

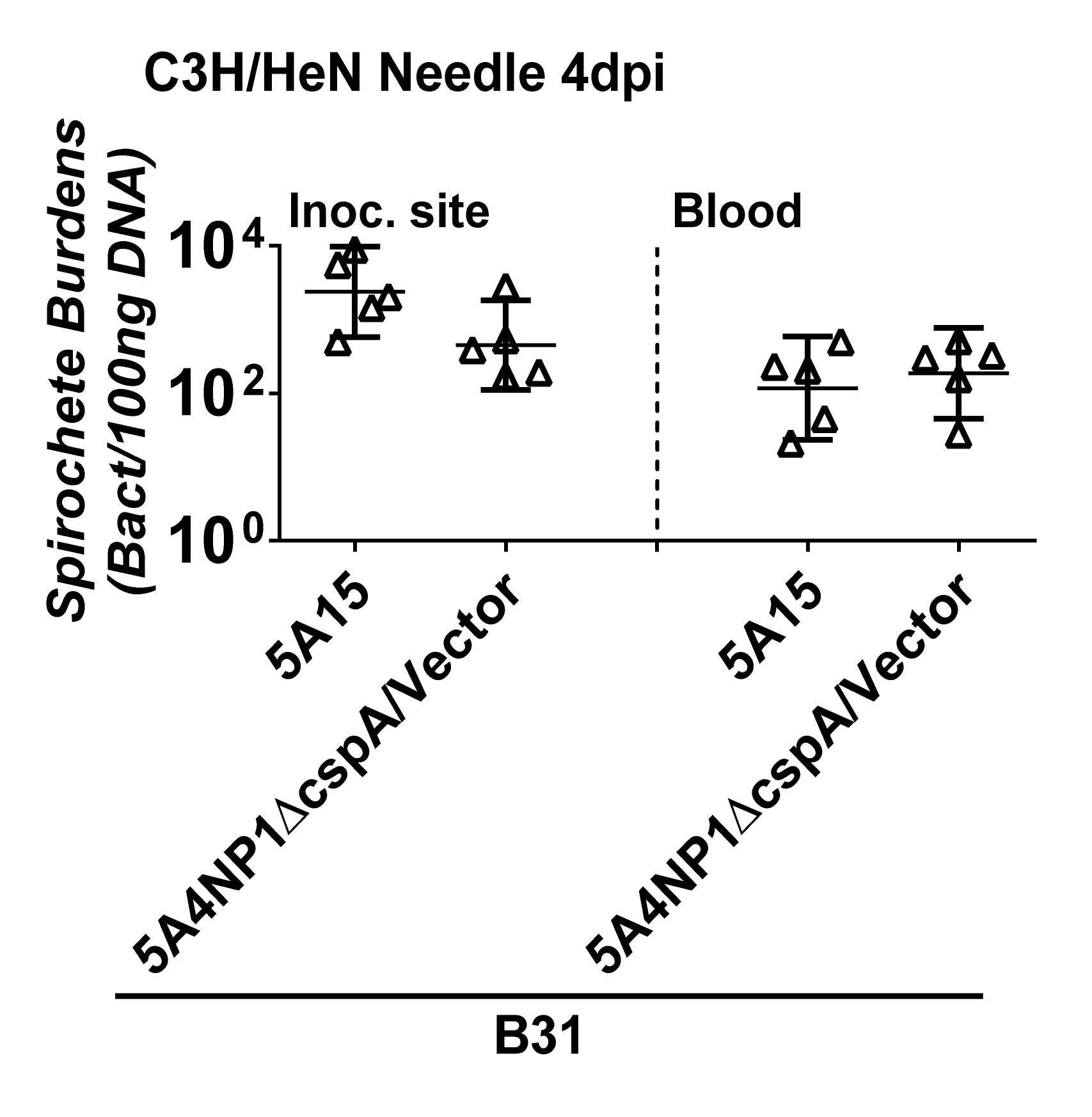

Supplement: S10 Fig — C3H/HeN mice were infected via needles with 105 B. burgdorferi strains B31-5A15 (“5A15”) or B31-5A4NP1ΔcspA harboring the vector pBSV2G (“5A4NP1ΔcspA/Vector”). At 4 days post infection (“4dpi”), the bacterial loads in the inoculation site of skin (“Inoc. Site”) and blood were determined by qPCR and normalized to 100 ng total DNA. Shown are the geometric mean of bacterial loads ± 95% confidence interval of 5 mice per group. No Significant differences (P > 0.05 by Student’s t test) of bacterial burdens were observed from the mice infected between these B. burgdorferi strains. (TIF) [file ppat.1007106.s010.tif]

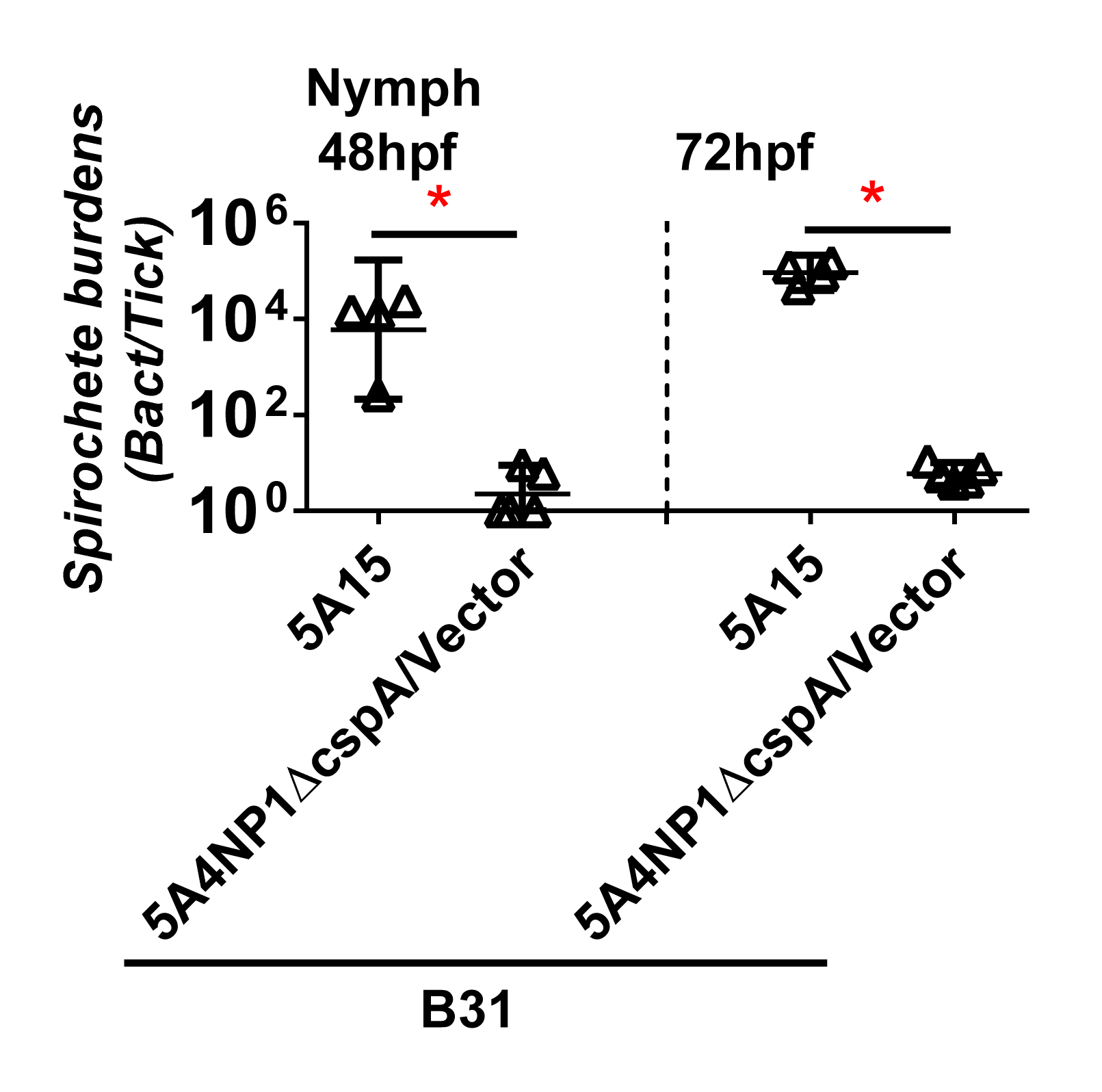

Supplement: S11 Fig — The nymphs infected with B. burgdorferi strain B31-5A15 (“5A15”) or a cspA mutant strain B31-5A4NP1ΔcspA harboring the vector pBSV2G (“5A4NP1ΔcspA/Vector”) were allowed to feed on C3H/HeN mice for 48 and 72 hours (“48hpf” and “72hpf”). Bacterial loads in those fed nymphs were determined by qPCR. Shown are the geometric mean of bacterial loads ± 95% confidence interval of four nymphs (for nymphs carrying strain B31-5A15) or five nymphs (for nymphs carrying the strain B31-5A4NP1ΔcspA harboring the vector) per group. “*” indicates statistically (P < 0.05 by Student’s t test) different between two different strains-infected nymphs. (TIF) [file ppat.1007106.s011.tif]

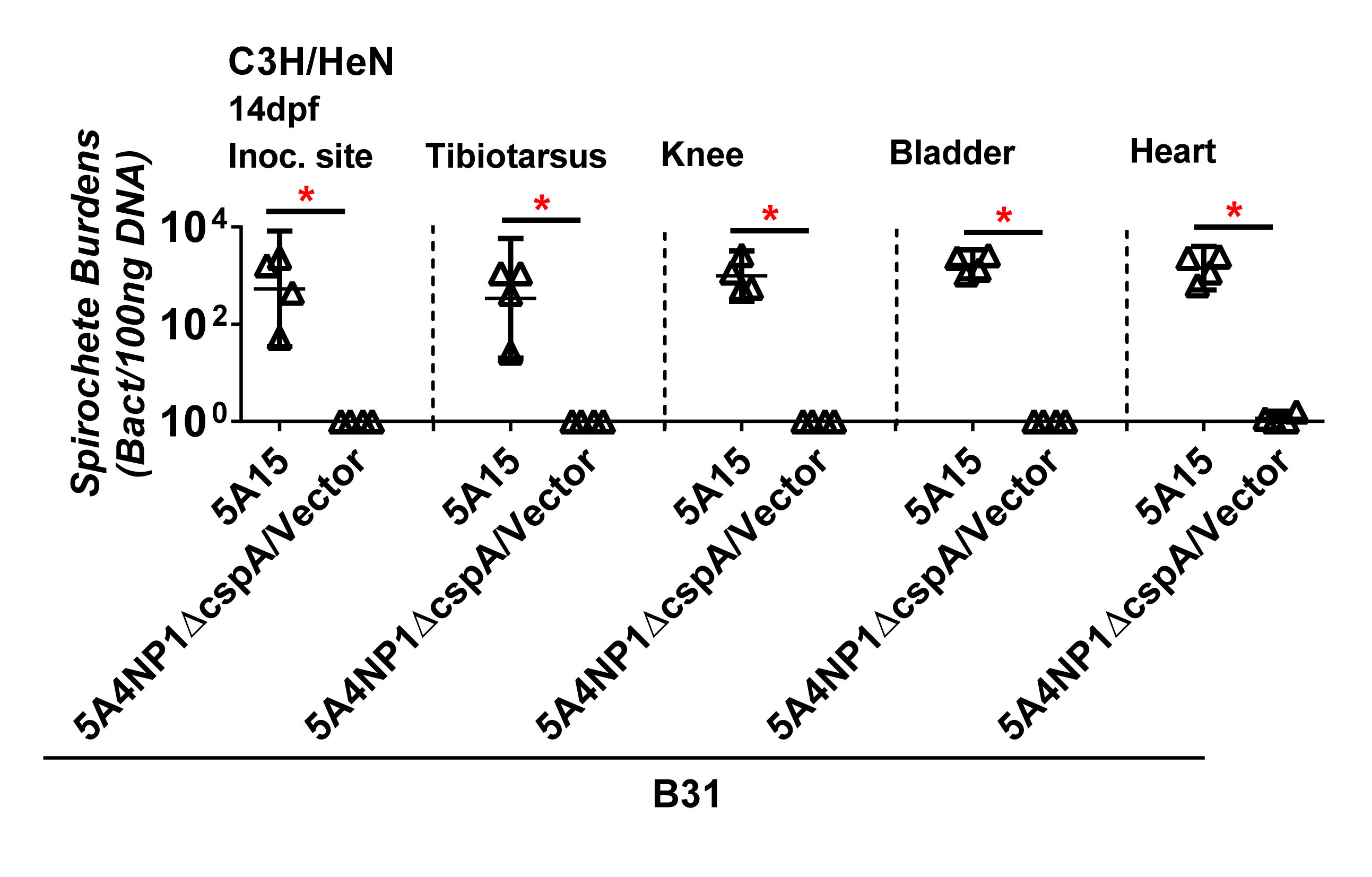

Supplement: S12 Fig — C3H/HeN mice were infected by needles with 105 B. burgdorferi strain B31-5A15 (“5A15”) or B31-5A4NP1ΔcspA harboring the vector pBSV2G (“5A4NP1ΔcspA/Vector”). At 14 days post infection, the uninfected I. scapularis larval ticks were allowed to feed on each of these mice to repletion. After the replete larvae molt into nymphs, those B. burgdorferi-infected nymphs were allowed to feed on naïve C3H/HeN mice to repletion. At 14 days after nymphal tick feeding (“14dpf”), the bacterial loads in the inoculation site of skin (“inno. site”), tibiotarsus and knee joints, bladder, and heart were determined by qPCR and normalized to 100 ng total DNA. Shown are the geometric mean of bacterial loads ± 95% confidence interval of four mice per group. Significant differences (P < 0.05, Student’s t test) in the spirochete burdens between two strains relative to each other (“*”). (TIF) [file ppat.1007106.s012.tif]

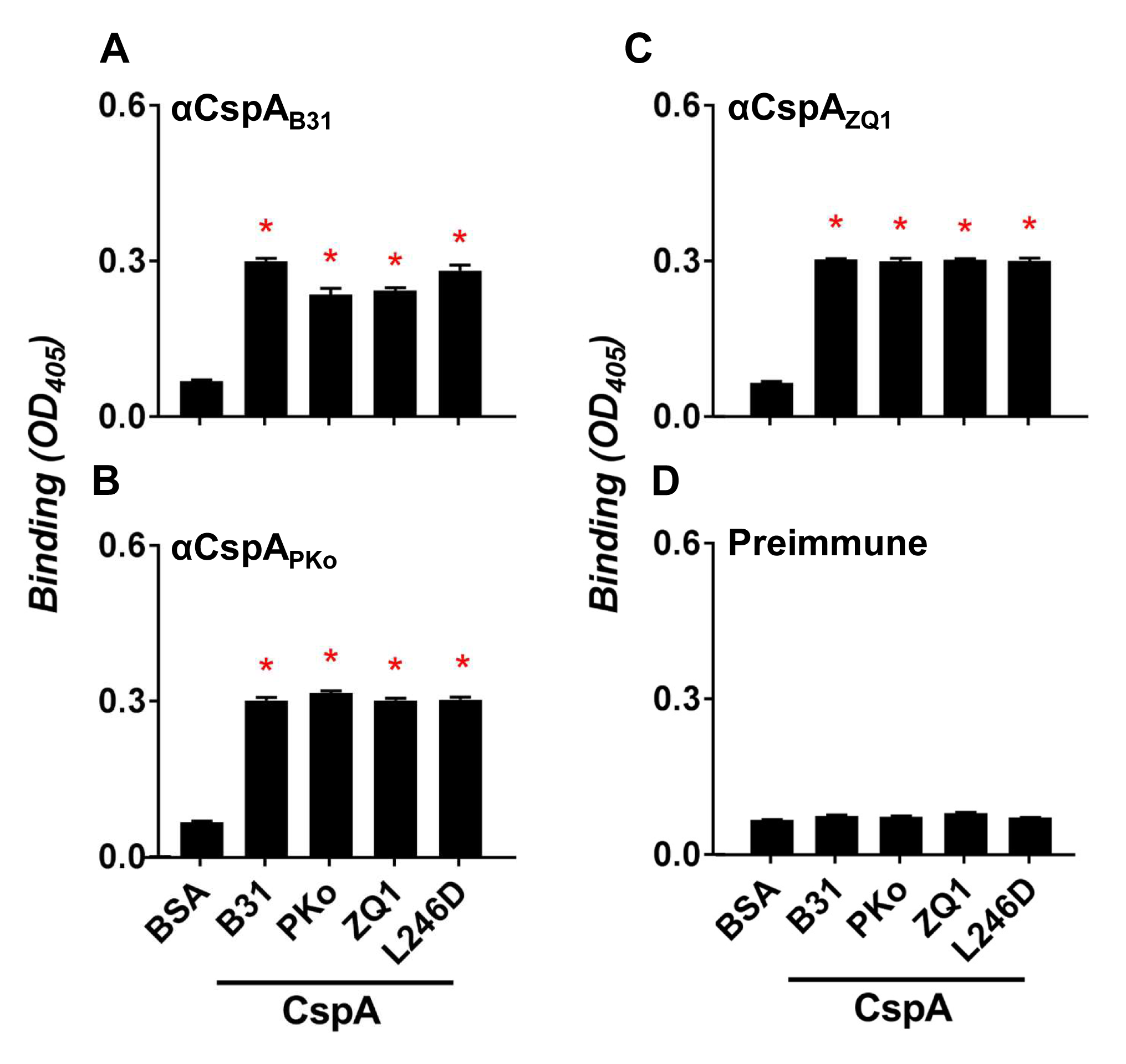

Supplement: S13 Fig — One microgram of CspAB31, CspAPKo, CspAZQ1, or CspAB31L246D, or BSA (as a negative control) was coated on triplicate wells in a microtiter plate. The anti-sera raised against (A) CspAB31 (“αCspAB31”), (B) CspAPKo (“αCspAPKo”), (C) CspAZQ1 (“αCspAZQ1”) or (D) pre-immune serum (“Preimmune”, negative control) were added to these wells. Bound antibody was measured by ELISA (see Materials and methods), and the mean OD405 values ± standard deviations were determined. Asterisk (“*”) indicates significant different levels of recognition by particular anti-sera to CspA proteins (P < 0.05) compared that to BSA determined by one-way ANOVA with post hoc Bonferroni correction. (TIF) [file ppat.1007106.s013.tif]
