# Supplementary material for: Polymorphic factor H-binding activity of CspA protects Lyme borreliae from the host complement in feeding ticks to facilitate tick-to-host transmission
Source: PLoS Pathog. 2018 May 29;14(5):e1007106. doi: 10.1371/journal.ppat.1007106 (PMC5993331; doi:10.1371/journal.ppat.1007106)
Supplement: S1 Table — (PDF) [file ppat.1007106.s014.pdf]

S1 Table. $CspA_{B31}$ and $CspA_{B31}$L246D display indistinguishable affinity in binding to C7, C9 and plasminogen.

		----- Surface Plasmon Resonance -----			
**Ligand**	**$CspA_{B31}$ protein**	**K_D (µM)**	k_{on} ($10^5 s^{-1} M^{-1}$)	k_{off} (s^{-1})	p^a
**C7**	**$CspA_{B31}$**	**5.26±1.57**	0.94±0.01	0.45±0.04	0.45
	**$CspA_{B31}$L246D**	**3.50±1.40**	1.43±0.04	0.38±0.02	
**C9**	**$CspA_{B31}$**	**5.47±0.66**	0.60±0.05	0.32±0.01	0.94
	**$CspA_{B31}$L246D**	**5.40±0.63**	0.70±0.01	0.36±0.01	
**Plasminogen**	**$CspA_{B31}$**	**6.35±1.02**	0.43±0.06	0.26±0.01	0.94
	**$CspA_{B31}$L246D**	**6.48±1.51**	0.49±0.01	0.29±0.03	

All values represent the mean ± SEM of three experiments.

[a]The statistical significance for the binding of a particular ligand to $CspA_{B31}$ or $CspA_{B31}$L246D was determined using T-test.
