# Supplementary material for: Polymorphic factor H-binding activity of CspA protects Lyme borreliae from the host complement in feeding ticks to facilitate tick-to-host transmission
Source: PLoS Pathog. 2018 May 29;14(5):e1007106. doi: 10.1371/journal.ppat.1007106 (PMC5993331; doi:10.1371/journal.ppat.1007106)
Supplement: S2 Table — (PDF) [file ppat.1007106.s015.pdf]

S2 Table. ***B. burgdorferi*** **strains and DNA plasmids used in this study**

Strain or plasmid	Genotype or characteristic	References or Sources
Borrelia strains		
ZQ1	Clonal isolate of *B. garinii* strain ZQ1	[76]
PKo	Clonal isolate of *B. afzelii* strain PKo	[25]
B313	High-passage *B. burgdorferi* B31 missing lp5, lp17, lp21, lp25, lp28-1, lp28-2, lp28-3, lp28-4, lp36, lp38, lp54, lp56, cp9, cp32-4, cp32-6, cp32-8, cp32-9	[24]
B313/pBSV2G	B313 carrying plasmid pBSV2G	This study
B31-5A15	Clonal isolate of *B. burgdorferi* strain B31 lacking lp21	[36]
B31-5A4NP1Δ*cspA*	B31-5A4NP1, the clonal isolate of strain B31 with *bbe02*:: KanR[a], *cspA*::StrR[b] , and lacking lp21	[28]
B31-5A4NP1Δ*cspA* /pBSV2G	B31-5A4NP1Δ*cspA* carrying plasmid pBSV2G	This study
B31-5A4NP1Δ*cspA* /pBSV2G-CspA$_{B31}$	B31-5A4NP1Δ*cspA* complemented with intact *cspA* (*bba68*) from *B. burgdorferi* strain B31 under the control of *cspA* promoter from this strain (PcspA).	This study
B31-5A4NP1Δ*cspA* /pBSV2G-CspA$_{PKo}$	B31-5A4NP1Δ*cspA* complemented with intact *cspA* (*bafPKo_A0067*) from *B. afzelii* strain PKo under the control of PcspA.	This study
B31-5A4NP1Δ*cspA* /pBSV2G-CspA$_{ZQ1}$	B31-5A4NP1Δ*cspA* complemented with intact *cspA* (*zqa68*) from *B. garinii* strain ZQ1 under the control of PcspA.	This study
B31-5A4NP1Δ*cspA* /pBSV2G-CspA$_{B31}$L246D	B31-5A4NP1Δ*cspA* complemented with intact *cspA* from *B. burgdorferi* strain B31 with leucine-246	This study

	replaced by aspartate under the control of PcspA.	
E. coli strains		
DH5α	F- Φ80lacZΔM15 Δ(lacZYA-argF) U169 recA1 endA1 hsdR17(rk-, mk+) phoA supE44 thi-1 gyrA96 relA1 λ-	ThermoFisher
BL21	F–, ompT, hsdSB (rB–, mB–), dcm, gal, λ(DE3)	Promega
M15 [Prep4]	F-, Φ80ΔlacM15, thi, lac-, mtl-, recA+ , KmR	Qiagen
BL21/pET30a-DbpA$_{B31}$	BL21 producing histidine tagged residue 26 to 192 of DbpA from *B. burgdorferi* strain B31	[77]
M15 [Prep4]/pQE30Xa-CspA$_{B31}$	M15 producing histidine tagged residue 26 to 252 of CspA (Bba68) from *B. burgdorferi* strain B31	This study
M15 [Prep4]/pQE30Xa-CspA$_{PKo}$	M15 producing histidine tagged residue 28 to 242 of CspA (BafPKo_A0067) from *B. afzelii* strain PKo	[25]
M15 [Prep4]/pQE30Xa-CspA$_{ZQ1}$	M15 producing histidine tagged residue 27 to 256 of CspA (Zqa68) from *B. garinii* strain ZQ1	This study
M15 [Prep4]/pQE30Xa-CspA$_{B31}$L246D	M15 producing histidine tagged residue 26 to 252of CspA (Bba68) from *B. burgdorferi* strain B31 with leucine-246 replaced by aspartate	This study
Plasmid		
pJET1.2/Blunt	AmpR^{c}; PCR cloning vector	ThermoFisher
pQE30Xa	AmpR^{c}; histidine-tag protein expression vector	Qiagen
pQE30Xa-CspA$_{B31}$	pQE30Xa encoding histidine fusion protein residue 26 to 252 of CspA (Bba68) from *B. burgdorferi* strain B31	This study
pQE30Xa-CspA$_{ZQ1}$	pQE30Xa encoding	This study

	histidine tagged protein residue 27 to 256 of CspA (Zqa68) from *B. garinii* strain B31ZQ1	
pQE30Xa-CspA$_{B31}$L246D	pQE30Xa encoding histidine fusion protein residue 26 to 251 of CspA (Bba68) from *B. burgdorferi* strain B31 with leucine-246 replaced by aspartate	This study
pBSV2G	GenR[d]; pBSV2-derived shuttle vector.	[82]
pBSV2G-CspA$_{B31}$	pBSV2G encoding intact *cspA* (*bba68*) from *B. burgdorferi* strain B31 under the control of *cspA* promoter from this strain (PcspA).	This study
pBSV2G-CspA$_{PKo}$	pBSV2G encoding intact *cspA* (*bafPKo_A0067*) from *B. afzelii* strain PKo under the control of pCspA	This study
pBSV2G-CspA$_{ZQ1}$	pBSV2G encoding intact *cspA* (*zqa68*) from *B. garinii* strain ZQ1 under the control of pCspA	This study
pBSV2G-CspA$_{B31}$L246D	pBSV2G encoding intact *cspA* (*bba68*) from *B. burgdorferi* strain B31 under the control of pCspA with leucine-246 replaced by aspartate	This study

KanR[a], Kanamycin resistant
StrR[b], Streptomycin resistant
AmpR[c], Ampicillin resistant
GenR[d], Gentamicin resistant
