# Supplementary material for: Polymorphic factor H-binding activity of CspA protects Lyme borreliae from the host complement in feeding ticks to facilitate tick-to-host transmission
Source: PLoS Pathog. 2018 May 29;14(5):e1007106. doi: 10.1371/journal.ppat.1007106 (PMC5993331; doi:10.1371/journal.ppat.1007106)
Supplement: S3 Table — (PDF) [file ppat.1007106.s016.pdf]

S3 Table. Primers used in this study.

Primer/Vector	Sequence*	Amplified DNA fragment
CspA$_{B31}$pfp/pQE30Xa	accGGATCCgcaccttttagc	*cspA$_{B31}$p*
CspA$_{B31}$prp/pQE30Xa	tttcGAATTCttagtaaaaggcaggttttaaag	
CspA$_{ZQ1}$pfp/pQE30Xa	cgGGATCCgttgataaaattgatcca	*cspA$_{ZQ1}$p*
CspA$_{ZQ1}$prp/pQE30Xa	cgGGATCCttaatagtcaagatttaa	
CspA$_{B31}$L246Dpfp/pQE30Xa	accGGATCCgcaccttttagc	*cspA$_{B31}$L246Dp*
CspA$_{B31}$L246Dprp/pQE30Xa	tcGTCGACttagtaaaaggcaggtttatcagtatc	
pcspA$_{B31}$fp/pBSV2G	cgGCATGCttacagctacaagaaaag	*pcspA$_{B31}$*
pcspA$_{B31}$rp/pBSV2G	cgGTCGACaatactctccttataaat	
CspA$_{B31}$fp/pBSV2G	cgGTCGACttgaaaaaagccaaacta	*cspA$_{B31}$*
CspA$_{B31}$rp/pBSV2G	cgGGATCCttagtaaaaggcaggttt	
CspA$_{PKo}$fp/pBSV2G	cgGTCGACttgacaaaaaccaacctg	*cspA$_{PKo}$*
CspA$_{PKo}$rp/pBSV2G	cgGGATCCctaattaatgggattaaa	
CspA$_{ZQ1}$fp/pBSV2G	cgGTCGACttgaaaaaaactaaactt	*cspA$_{ZQ1}$*
CspA$_{ZQ1}$rp/pBSV2G	cgGGATCCctaattaatgggattaaa	
CspA$_{B31}$L246Dfp/pBSV2G	cgGTCGACttgaaaaaagccaaacta	*cspA$_{B31}$L246D*
CspA$_{B31}$L246Drp/pBSV2G	tcGGATCCttagtaaaaggcaggtttatcagtatc	
BBCspAfp	cttaaacgaaactcttaaagtttacaatca	*BBcspA$_{B31}$*
BBCspArp	ttagtaaaaggcaggttttaaagtatcaaa	
BBFlaBfp	gcagctaatgttgcaaatcttttc	*BBflab*
BBFlaBrp	gcaggtgctggctgttga	
BBRecAfp	gtggatctattgtattagatgaggctctcg	*BBrecA*
BBRecArp	gccaaagttctgcaacattaacacctaaag	

ColE1fp	ctacatacctcgctctgctaatc	*BBcolE1*
ColE1rp	cgaaacccgacaggactataaa	
mNidfp	ccagccacagaatcccatcc	*mNidogen*
mNidrp	ggacatactctgctgccatc	
Kanfp	atgagccatattcaacgggaa	Kanamycin
Kanrp	ttagaaaaactcatcgagcat	
Genfp	atgttacgcagcagcaac	Gentamycin
Genrp	ttaggtggcggtacttgg	
Strfp	caggatgacgcctaacaa	Streptomycin
Strrp	ccaccttcaacagatcgc	

* Restriction sites used are shown in capital letters.
